# Combination of verteporfin-photodynamic therapy with 5-aza-2’-deoxycytidine enhances the anti-tumour immune response in triple negative breast cancer

**DOI:** 10.3389/fimmu.2023.1188087

**Published:** 2023-11-07

**Authors:** Shramana M. Banerjee, Pilar Acedo, Soha El Sheikh, Rania Harati, Amelia Meecham, Norman R. Williams, Gareth Gerard, Mohammed R. S. Keshtgar, Alexander J. MacRobert, Rifat Hamoudi

**Affiliations:** ^1^ Breast Unit, Royal Free London National Health Service (NHS) Foundation Trust, London, United Kingdom; ^2^ Division of Surgery and Interventional Science, University College London, London, United Kingdom; ^3^ Institute for Liver and Digestive Health, Division of Medicine, University College London, London, United Kingdom; ^4^ University College London (UCL) Cancer Institute, University College London, London, United Kingdom; ^5^ Department of Pharmacy Practice and Pharmacotherapeutics, College of Pharmacy, University of Sharjah, Sharjah, United Arab Emirates; ^6^ Research Institute for Medical and Health Sciences, College of Medicine, University of Sharjah, Sharjah, United Arab Emirates

**Keywords:** verteporfin, photodynamic therapy, 5-aza-2’-deoxycytidine, anti-tumour immune response, triple negative breast cancer, 4T1

## Abstract

**Introduction:**

Triple negative breast cancer (TNBC) is a subtype of breast cancer characterised by its high tumourigenic, invasive, and immunosuppressive nature. Photodynamic therapy (PDT) is a focal therapy that uses light to activate a photosensitizing agent and induce a cytotoxic effect. 5-aza-2’-deoxycytidine (5-ADC) is a clinically approved immunomodulatory chemotherapy agent. The mechanism of the combination therapy using PDT and 5-ADC in evoking an anti-tumour response is not fully understood.

**Methods:**

The present study examined whether a single dose of 5-ADC enhances the cytotoxic and anti-tumour immune effect of low dose PDT with verteporfin as the photosensitiser in a TNBC orthotopic syngeneic murine model, using the triple negative murine mammary tumour cell line 4T1. Histopathology analysis, digital pathology and immunohistochemistry of treated tumours and distant sites were assessed. Flow cytometry of splenic and breast tissue was used to identify T cell populations. Bioinformatics were used to identify tumour immune microenvironments related to TNBC patients.

**Results:**

Functional experiments showed that PDT was most effective when used in combination with 5-ADC to optimize its efficacy. 5-ADC/PDT combination therapy elicited a synergistic effect *in vitro* and was significantly more cytotoxic than monotherapies on 4T1 tumour cells. For tumour therapy, all types of treatments demonstrated histopathologically defined margins of necrosis, increased T cell expression in the spleen with absence of metastases or distant tissue destruction. Flow cytometry and digital pathology results showed significant increases in CD8 expressing cells with all treatments, whereas only the 5-ADC/PDT combination therapy showed increase in CD4 expression. Bioinformatics analysis of in silico publicly available TNBC data identified BCL3 and BCL2 as well as the following anti-tumour immune response biomarkers as significantly altered in TNBC compared to other breast cancer subtypes: GZMA, PRF1, CXCL1, CCL2, CCL4, and CCL5. Interestingly, molecular biomarker assays showed increase in anti-tumour response genes after treatment. The results showed concomitant increase in BCL3, with decrease in BCL2 expression in TNBC treatment. In addition, the treatments showed decrease in PRF1, CCL2, CCL4, and CCL5 genes with 5-ADC and 5-ADC/PDT treatment in both spleen and breast tissue, with the latter showing the most decrease.

**Discussion:**

To our knowledge, this is the first study that shows which of the innate and adaptive immune biomarkers are activated during PDT related treatment of the TNBC 4T1 mouse models. The results also indicate that some of the immune response biomarkers can be used to monitor the effectiveness of PDT treatment in TNBC murine model warranting further investigation in human subjects.

## 1 Introduction

Breast cancer is a chronic complex disease and a leading cause of cancer death amongst women globally (1). It is a heterogeneous disease classified into different molecular subtypes based on the presence (+) or absence (-) of immunohistochemical markers such as ER (estrogen receptor), PR (progesterone receptor), and HER2/neu (human epidermal growth factor receptor 2) in the breast tumour. Initially, Breast Cancer (BC) was classified based on hormonal receptors (ER and PR) alone. Later, new techniques developed identified HER2 as an additional marker (2). One of the subtypes is triple-negative breast cancer (TNBC) which is characterized by negative expression for ER, PR, and HER2 (ER-, PR-, and HER2-) and poor patient survival (3). The conventional treatment of primary breast cancer centres on surgery and adjuvant treatments including systemic chemotherapy, radiotherapy, endocrine therapy, and targeted therapies. The improvements made in therapy regimens over the last 20 years have reduced the mortality rate and improved morbidity among patients (4). However, despite the major advances made by systemic therapy regimens, novel therapies with enhanced efficacy and fewer side effects are required to treat patients presenting with TNBC.

Photodynamic therapy (PDT) is a site-specific, minimally invasive, and clinically approved treatment based on a photochemical reaction between light, a light-activated molecule or photosensitiser (PS) and molecular oxygen to elicit cell death (5, 6). The procedure consists of the pre-administration of a PS and its uptake by malignant cells, followed by local illumination of the target lesion with visible light at a specific wavelength (7–9). Each individual component is harmless, but the combination results in the formation of reactive oxygen species (ROS) that damages key components associated with the cell membrane, cytoskeleton, mitochondria, endoplasmic reticulum and nucleic acids, among others, which promotes immunogenic apoptosis or necrosis of malignant cells depending on the light treatment dose (10–12). In addition to its direct cytotoxic effect on malignant cells, PDT can also damage the tumour vasculature (13–16) and is shown to induce inflammatory and anti-tumour innate and adaptive immune responses capable of eradicating distant untreated tumour cells and developing anti-tumour memory immunity that can potentially suppress metastasis and prevent cancer recurrence (17). Indeed, cell death induced by PDT is accompanied by the release and/or exposure of damage-associated molecular patterns (DAMPs) by the dying cells. This triggers recruitment of antigen presenting cells (APC) such as monocytes or macrophages, neutrophils and dendritic cells to the site of cellular injury to eliminate photo-damaged tumour cells by phagocytosis. Subsequently, the recruited immune cells present antigen-derived peptides in association with major histocompatibility complex (MHC) molecules to T lymphocytes which results in activation of CD4+ T helper cells, CD8+ cytotoxic T cells as well as B cells, and hence the initiation of an adaptive immunity eventually allowing the control of distant metastases and protection from tumour relapse (17–24).

One promising approach to enhance PDT effectiveness against malignant cells consists of combining PDT with other therapeutic modalities, notably with surgery, radiotherapy, chemotherapy and immunotherapy (25–28). These combination regimens may potentiate the anti-tumour effect as well as the immune response. In this context, PDT combined with immunomodulatory agents showed improved efficacy in tumour cell destruction, stasis in tumour growth and induction of a sustained immune response in a number of cancer mouse models including breast cancer (26, 28–31). The optimization and a better understanding of such multi-modal regimens combining PDT with other anti-tumour therapies notably with immunomodulatory agents may lead to complete tumour eradication and long-term control of distant metastases.

5-aza-2′-deoxycytidine (5-ADC) is an immunoregulatory agent clinically approved for the treatment of myelodysplastic syndrome and acute myeloid leukaemia and is currently under investigation for treatment of advanced solid tumours (32, 33). 5-ADC is a methyltransferase inhibitor that inhibits DNA methylation, an epigenetic mechanism that regulates gene transcription (34). In cancer cells, aberrant DNA methylation plays an important role in tumourigenesis, silencing of tumour suppressor genes, as well as evasion of tumour cells from immune surveillance (35–37). In breast cancer cells, DNA methylation represses expression of MHC molecules as well as tumour antigens needed to induce an anti-tumour immune response. Inhibition of DNA methylation by 5-ADC restores expression of tumour antigens and MHC molecules on breast cancer cells and leads to better antigen presentation and subsequently the recognition of tumour cells by cytotoxic T lymphocytes and the initiation of an anti-tumour immune response (38). Taken together, these data suggest that combining PDT with the immunomodulatory agent 5-ADC could improve the efficacy of PDT and enhance its anti-tumour immune response.

PDT has proven to be an effective anti-tumour treatment (7–9) and has been investigated as a primary clinical treatment in a number of solid tumours. Clinical studies in pancreatic, prostate, head and neck tumours showed that PDT is successful and well tolerated with few adverse effects (39–41). In a first clinical study of PDT in the treatment of primary breast cancer, we showed that PDT under image guidance was a promising and safe therapeutic option in a multi-therapy setting in patients with a poor or incomplete response to neoadjuvant therapy (25). In this study we used verteporfin in a liposomal formulation (Visudyne™) as the photosensitiser (verteporfin-PDT). Verteporfin is a second-generation clinically approved PS for the treatment of age-related macular degeneration. We employed the name verteporfin throughout the manuscript as is common practice in the literature. In addition, clinical studies have demonstrated the anti-tumour efficacy and overall safety of verteporfin-PDT for the treatment of locally advanced pancreatic cancer (8, 42). Verteporfin is particularly suitable for use in breast cancer therapy due to its liposomal formulation (Visudyne™) allowing improved drug delivery, absorption, permeability and retention in tumour vasculature (43). While verteporfin-PDT has shown numerous advantages, more research is still needed to improve the anti-tumour efficacy of PDT.

The present study’s aim is to examine whether a single dose of the immunomodulatory drug, 5-ADC, enhances the cytotoxic and anti-tumour immune effects of low dose PDT in primary breast cancer using a liposomal formulation of verteporfin as the PS. In addition, the study aimed to examine the optimum conditions of the combination therapy of verteporfin-PDT and 5-ADC in terms of light dose, verteporfin and 5-ADC concentrations *in vitro* and *in vivo* in an orthotopic breast cancer mouse model using the triple negative mouse mammary tumour cell line 4T1 known to be highly tumourigenic, invasive and immunosuppressive (44, 45), which mirrors the clinical sequelae associated with triple-negative human breast cancer (46, 47).

## 2 Materials and methods

### 2.1 Cell culture

The unmodified 4T1 mouse mammary tumour cell line was obtained from Caliper Life Sciences, United Kingdom, and cultured in RPMI 1640 Medium supplemented with 10% FBS and antibiotics (100 U/mL penicillin and 0.1 mg/mL streptomycin). Cells were routinely cultured in 75cm^2^ tissue culture flasks (TPP) at 37°C in 95% humidity and 5% CO_2_ incubator. Once the flasks reached a 70-80% confluency, cells were either sub-cultured or seeded for the desired protocols. When necessary, cells were detached using a solution of 0.05% Trypsin-EDTA.

### 2.2 Pharmacological drugs

5-aza-2′-deoxycytidine (5-ADC) was purchased from Sigma Aldrich Ltd (United Kingdom). Verteporfin in a liposomal formulation (Visudyne™) was purchased from Novartis^©^ (United Kingdom) via the Royal Free London Pharmacy and was used as PS in this study. Both drugs were reconstituted with sterile distilled water.

### 2.3 *In vitro* treatments

To determine the half maximal inhibitory concentration (IC50) of 5-ADC, 4T1 cells (3×10^3^ cells/well) were seeded in a 96-well plate. After 24 h, cells were washed once with PBS then treated with increasing doses of the drug ranging from 0.1 μM to 1 μM for 24h. Cells exposed to the drug for 24 hours were then washed twice with PBS to remove all of the 5-ADC and allowed to grow for a further 48h. Cell viability was then assessed using the 3-(4,5-dimethylthiazol-2-yl)-2,5-diphenyltetrazolium bromide assay (MTT assay), as explained below.

To determine the lowest optimum light dose and concentration of verteporfin needed to achieve 50% cell growth inhibition, 4T1 cells (3× 10^3^ cells/well) were seeded in 96-well plates. 1 J/cm^2^ and 2.5 J/cm^2^ were selected as the test light doses based on previous work done by the group and published articles. Twenty-four hours after plating, cells were treated with increasing concentrations of verteporfin ranging from 0.1 μM to 0.5 μM. The plates were covered with aluminium foil to minimize inadvertent photosensitization. After 90 minutes, cells were washed twice with PBS then exposed to either 1 J/cm^2^ or 2.5 J/cm^2^ of red light using a filtered xenon arc lamp coupled to a light guide (integrated with a lens to generate a uniform output beam) centred at 690 +/- 15 nm (GCG Healthcare Ltd, West Sussex, UK). The intensity of the lamp was measured prior to irradiation and was calculated to be 20mW/cm^2^. Activation of verteporfin occurs using near infra-red light peaking at wavelength 690 nm (43). Following illumination, the plate was re-covered with aluminium foil and placed in the incubator for a further 48h. Changes in proliferation rate or metabolic activity were then assessed with MTT assay. A treated but not illuminated plate was also included in our studies to validate the lack of toxicity induced by verteporfin in our model in the dark, as previously observed for other cancers.

For the combinatorial treatment and to determine the optimum concentration of verteporfin combined with 5-ADC that results in maximal response, 4T1 cells (3×10^3^ cells/well) were seeded in a 96-well plate and allowed to proliferate for 24h. Cells were then washed and treated with 5-ADC (0.5 µM) for 24h. After 24h, cells were treated with liposomal formulation of verteporfin for 90 minutes then washed and illuminated. Concentrations tested at 1J/cm^2^ and 2.5 J/cm^2^ were 0.25 μM and 0.15 μM verteporfin, respectively, based on the results obtained in the monotherapy studies. After illumination, the plates were kept in the incubator as previously described for 48h. Then metabolic activity was assessed by the MTT assay. Three independent experiments were performed.

### 2.4 Cell proliferation assay

Cell proliferation or metabolic activity was evaluated using the 3-(4,5-dimethylthiazol-2-yl)-2,5-diphenyltetrazolium bromide assay (MTT assay). 100 μL of MTT (1 mg/ml) was prepared in RPMI, filtered via a 0.22 μm PVDF syringe filter, and added to each well. Cells were incubated for 1 h. Supernatant was then removed and 100 μL of DMSO (dimethyl sulfoxide) was added to each well to dissolve the formazan crystals. Absorbance was measured at 570 nm using an Infinite M200 PRO microplate reader (Tecan Group Ltd., Switzerland). Three independent experiments were performed (six wells per condition and experiment).

### 2.5 Western blot

For protein extraction, 4T1 cells (100000 cells/well) were grown in 6-well plates and treated as previously explained. Forty-eight hours post-treatment, cells were washed with PBS and lysed with RIPA lysis buffer supplemented with COMPLETE protease inhibitor cocktail (Roche). Afterwards, cells were centrifuged at 14,000rpm for 20 minutes at 4°C, and protein concentration of all supernatants was measured using the commercial Bio-Rad Bradford protein assay. Protein electrophoresis was done by standard SDS-PAGE using 4-20% polyacrylamide gels and transferred to PVDF membranes (BioRad). Blocking was performed with 5% semi-skimmed milk (diluted in PBS) for 1 hour at room temperature. Membranes were incubated overnight at 4°C with primary antibodies diluted in 5% semi-skimmed milk against Dnmt1 (DNA (cytosine-5)-methyltransferase 1) (1:1000, ab188453, Abcam); YAP (yes-associated *protein* 1) (1:500, sc-101199, Santa Cruz Biotechnology); Cleaved caspase-3 (Asp175) (1:1000, 9661, cell signalling) and GAPDH (Glyceraldehyde 3-phosphate dehydrogenase) (1:10,0000, sc-32233, Santa Cruz Biotechnology). Membranes were then washed thrice for 5 minutes with TBS 0.1% Tween-20 and incubated with goat anti-mouse or anti-rabbit HRP-linked secondary antibodies (1:10,000; P0447 and P0448, Dako) for 1 hour at room temperature. Membranes were developed using the western-Ready ECL Substrate solution (BioLegend) and a ChemiDoc XRS+ gel imaging system (Bio-Rad). The relative intensity of the bands was analysed by Image J (National Institute of Health, USA).

### 2.6 Immunofluorescence

For indirect immunofluorescence detection of cleaved caspase 3 and YAP, 4T1 cells grown on coverslips in a 24-well plate at a cell density of 25,000 cells/well in 500 μL of RPMI. Post-treatment, cells were fixed for 30 minutes with 4% paraformaldehyde in PBS at RT, washed three times with PBS (5 min each), and permeabilized with 0.5% Triton X-100. After 5 min, Triton X-100 was removed and cells were incubated in blocking solution (5% bovine serum albumin, 5% FBS, 0.02% Triton X-100 in PBS) for 30 min at RT. Once removed from blocking solution, 25 μl of a 1:100 solution of primary antibody (same ones used for western blotting) was added to each sample and incubated at 37 °C for 1 h. Three 5-min washes with PBS were then carried out before addition of Triton X-100 for 5 min. Incubation with Alexa Fluor 488 goat anti-mouse or anti-rabbit IgG secondary antibody (1:500) was identical to that of the primary antibody and so were final washes. Samples were mounted with ProLong Gold antifade with 4,6-diamidino-2-phenylindole (DAPI) to stain the nuclei. Samples were visualised using an Olympus BX63 microscope with a digital camera DP80, equipped with the filters: blue/DAPI (350-460 nm) and green/FITC (495-524 nm). Images were merged using ImageJ software (National Institute of Health, USA).

### 2.7 Orthotopic mouse model using 4T1 cells

The animal experiments were undertaken under the ethical approval of the project (REC approval number: 70/7666) and personal licenses (PIL70/25583) granted by UK Home Office (2013) and adhering to the United Kingdom Coordinating Committee of cancer Research (UKCCCR) guidelines. Six-week-old immuno-competent Balb/c female mice were purchased from Worthington laboratories. At 8 weeks age, 36 mice were inoculated into the abdominal mammary pad with 4x10^5^ 4T1 cells suspended in RPMI medium. Once tumours were first palpable (5 days after inoculation with tumour size between 6 and 10 mm), verteporfin-PDT alone (n=9) (15mg/kg), 5-ADC alone (6.25mg/Kg) (n=8), or for the combination strategy, single dose 5-ADC (6.25mg/Kg) followed 48h later with verteporfin-PDT (n=9). Control mice (n=10) were inoculated with tumour cells but untreated (only saline was injected). The extent of tumour damage induced by treatment was assessed histologically, as described later. 5-ADC was injected intraperitoneally, while verteporfin-PDT was performed following intravenous injection of verteporfin using the clinically approved liposomal formulation (Visudyne™) (15mg/Kg) via the tail vein. A fibre-optically coupled laser diode module emitting at 690 nm (Biolitec AG) was used for PDT and activated verteporfin at its 690nm absorption peak. The power employed did not exceed 100mW. Before PDT treatment, mice were given general anaesthesia and the treatment light dose was delivered either 15 minutes or 60 minutes after verteporfin intravenous injection via a bare-end optical fibre (Medlight SA, Switzerland) introduced directly into the tumour’s superior pole, via an excision in the overlying skin. Following PDT treatment, the laser fibre was removed, and the wound closed and disinfected with chlorhexidine. The treated mice were given buprenorphine via intra-muscular injection and acetaminophen for analgesia. Mice were initially kept in a dark, humidified, and warm environment for recovery, and then returned to their normal cages at room temperature. Mice were observed daily and biometric measurements, including tumour diameter, were performed. Mice were also observed daily for further symptomatic deterioration or behavioural change suggestive of increasing pain and requiring euthanasia in accordance with home office guidelines. Mice were sacrificed 5 days after PDT and no later than day 14 after inoculation of tumour cells.

### 2.8 Identification of tumour immune microenvironment response genes using bioinformatics and *in silico* analysis

In order to identify the immune response genes to be tested for, thorough literature search was carried to identify immune response gene linked to breast cancer. The expression of each gene in TNBC was tested for significant by comparing 293 TNBC samples to 3887 of non-TNBC subtypes of breast cancer in human samples using bc-GenExMiner bioinformatics tool (http://bcgenex.ico.unicancer.fr) (48). To characterise the potential the relationships between the significantly differentially expressed panel of genes in TNBC compared to non-TNBC, the STRING database (49) was used to generate graph-theoretic protein-protein interaction map from the panel.

### 2.9 Quantitative real-time PCR

Total RNAs were extracted from cells (*in vitro* studies) or from formalin fixed paraffin embedded (FFPE) tissue blocks from breast and splenic murine tissues according to standard procedures. Quantitative real-time PCR (qRT-PCR) was performed using the QuantStudio 3 Real-Time PCR System (Thermofisher) and SYBR Green chemistry (Qiagen Quantifast SYBR Green kit (Cat# 204056, Qiagen)). The forward and reverse primers were designed or modified from DuPré et al. (50) to work on FFPE biopsies. The list of primer sequences is presented in **Supplementary Table 1**. GAPDH was used as the internal standard housekeeping gene. The qPCR data was analysed using the QuantStudio Design and Analysis Software v1.4.1 (ThermoFisher). The relative gene expression was determined using the 2^-ΔCt^ and 2^-ΔΔCt^ methods (51). Data are presented as the mean of three independent experiments performed in duplicates.

### 2.10 Flow cytometry

Splenic tissue harvested for flow cytometry was dissected, digested and then re-suspended in FACS buffer solution (Invitrogen). Fc/32 block antibodies were added to the solution to decrease non-specific binding of immunoglobulins and incubated for 30 minutes at 4°C in the dark. Primary antibodies for CD4 (L3t4) biotin, CD8 (PE/Cyanine7 anti-mouse CD8a antibody (ly-2)), MHC class II (I-A/I-E), CD16/32 APC monoclonal antibody and CD45 antibody-FITC were then added and incubated for 45 minutes. Controls were provided by adding a drop of UltraComp eBeads in FACS buffer in each of the 4 antibodies individually. Quantitative data from flow cytometry samples were analysed by setting the flow cytometer (FortessaTM) threshold for forward angle scatter (FS) and 4 colours of fluorescence were collected using logarithmic amplification as follows: PE (670nm, for CD8+), FITC (520nm for MHC class II+), BV 510 (510nm for CD45+), and BV 421(421nm for CD4+). Gating and further analysis was carried out using the FlowJo v10 software and was based on dead cells debris exclusion of material with minimal forward or side scatter from the analysis and CD45+ staining to identify the percentage of CD4+ and CD8+ lymphocytes in the sample. Thus, the gating strategy was to identify the percentage lymphocytes in the sample based on side scatter versus forward scatter plots then identifying the percentage CD4+ and CD8+ within the gated region.

### 2.11 Histopathology analysis

The breast tumours and spleens of all mice were harvested at post-mortem and placed in 10% formal saline. All specimens were processed for histopathology analysis within 24 hours of retrieval and routinely processed (CI. Biobank for health and disease UCL). H&E (hematoxylin and eosin) stained serial sections were examined to assess the size of breast tumours, and the extent of confluent tumour necrosis both at the peripheral PDT sites and centrally within the tumour. Necrosis refers to zonal replacement of viable tissue by amorphous eosinophilic material with ghost outlines and karyorrhectic debris. Individual cell apoptosis was not considered. The tumour diameter and maximum necrotic diameter (within the tumour) were used to calculate an estimate for tumour and necrosis volume using the formula: V = 4/3 π (D/2)^3^ where V is the volume, π is 3.14 and D is the measured diameter.

Spleens were assessed for the presence of metastases. In addition, pathological changes to the normal architecture to suggest tumour invasion, increase in immunological activity were also recorded. Immunohistochemistry, using anti-CD4 (ab183685, Abcam, 1:500 dilution) and anti-CD8 (ab209775, Abcam, 1:1000 dilution) antibodies, was employed to identify the presence of CD 4+ helper T cells, CD 8+ cytotoxic T cells, to characterize the 4T1 cells MHC class II in the treated versus control splenic tissue. Lung, liver, and breast tissues were assessed for the presence of metastatic tumour cells.

### 2.12 Quantitative analysis using digital pathology

In order to detect the effect of PDT on tumour tissue at the cellular level, digital pathology techniques were implemented using ImageJ plugin ‘IHC profiler’ (52). The method uses colour deconvolution and pixel profiling to produce a score for images with IHC staining using the following formula: $\text{Score}=\;\frac{\left(\text{No of pixels in an area}\right)\left(\text{Score of the area}\right)}{\text{Total No of pixels in the image}}$ .

For the splenic red and white pulp quantification, the area of each was quantified. For the immunohistochemistry analysis, the staining area (percentage) and intensity for each antibody was estimated. The images were cropped by setting the scale and creating an image with the same dimension across the different slides and the proportion score (PS) and an intensity score (IS) were calculated as described in Berryman et al. (52) which produced the criteria used for quantitative digital pathology (**Table 1**).

**Table 1:** Digital pathology criteria for quantitative analysis based on proportion and intensity scores.

Percentage of positivity	Proportion score (PS)	Intensity score (IS)
0%	0	Negative 0
1–10%	1	Low positive 1
11–50%	2	Positive 2
51–80%	3	High positive 3
81–100%	4	Substantial positive 4

### 2.13 Statistical analysis

Statistical analyses were carried out using two-way ANOVA (analysis of variance) followed by Bonferroni *post hoc* test for multiple comparisons. p<0.05 was considered to be statistically significant and at probability levels of p<0.05(*), p<0.01(**), and p<0.001(***). Calculations and figures were generated using GraphPad-Prism software (version 8.2.0). Data are shown as mean ± SD (standard deviation) from three independent experiments (biological replicates).

For digital pathology, Fisher’s exact test was used to compare untreated and various treated groups. For qRT-PCR, the data were calculated as the average from three independent experiments. The fold change was calculated by comparing each of the treatment groups (verteporfin-PDT, 5-ADC and verteporfin-PDT with 5-ADC) against the control (untreated) and presented as fold change for each of the gene being investigated.

The synergistic interaction between verteporfin-PDT and 5-ADC used in combination was assessed by applying the method of Valeriote and Lin (53–55) as follows:

[A]: % of cell viability after treatment A (verteporfin-PDT 0.15 µM at 1 J cm^-2^ or 0.25 µM at 1 J cm^-2^ in monotherapy), [B] % of cell viability after treatment B (0.5 µM 5-ADC in monotherapy), and [A + B] % cell viability after combination treatment (verteporfin-PDT+5-ADC). The effect of the combination treatment is defined as:


Synergistic if:[A+B]<[A] x [B]/100 or

Additive if:[A+B]=[A] x [B]/100

## 3 Results

### 3.1 *In vitro* combination therapy of verteporfin-PDT and 5-ADC exerts synergistic cytotoxic effect on 4T1 TNBC cells

**Figure 1:**
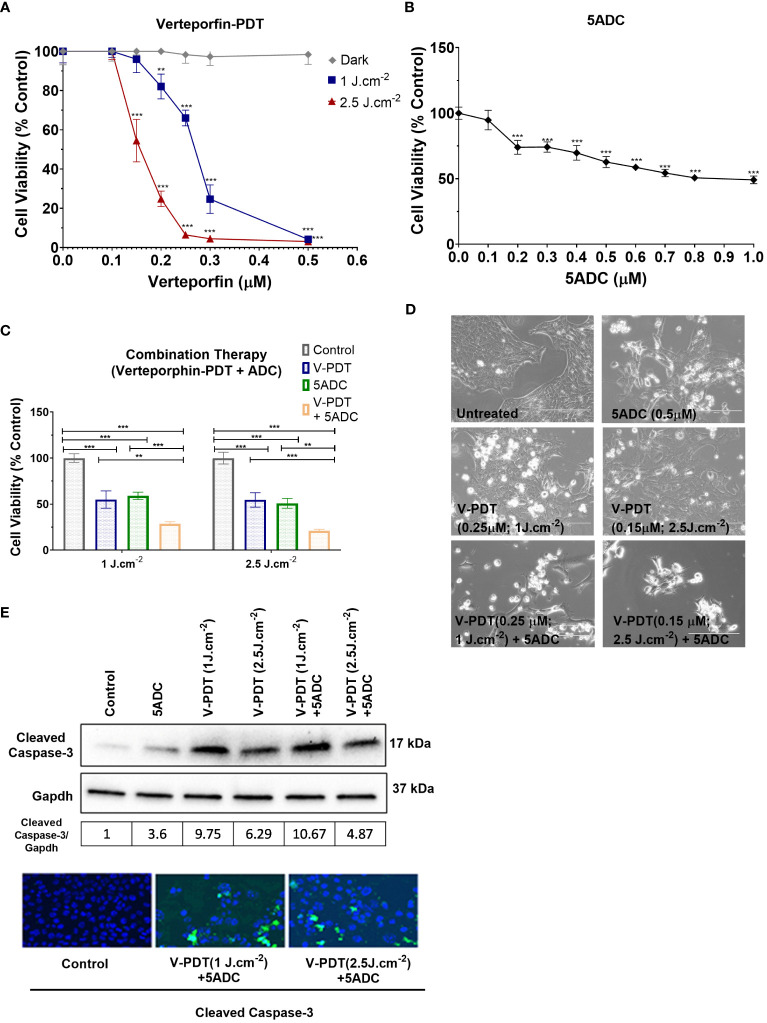
Effects of the combination of verteporfin-PDT with 5-ADC on viability of 4T1 TNBC cell line. **(A)** 4T1 cell line was seeded in 96-well plates (3 × 10^3^ cells per well) and allowed to proliferate for 24 h in complete medium. After 24h, the cells were incubated for 90 min in the presence or not of verteporfin at different concentrations. After 90 minutes of incubation, the cells were washed and exposed to either 1 J cm^-2^ or 2.5 J cm^-2^ of red light. Following illumination, the plate was re-covered with aluminium foil and placed in the incubator. At 48 hours of further incubation cell viability was assessed with MTT assay. Data are mean - SD of three independent experiments performed in sextuplicates. **(B)** 4T1 breast cancer cell line was seeded in 96-well plates (3 × 10^3^ cells per well) and allowed to proliferate for 24 h in complete medium. Then cells were incubated for a further 48 h in the presence or not of 5-ADC at different concentrations. At the end of the treatment period, cell proliferation was measured using the MTT assay. Data are mean +/- SD of three independent experiments performed in sextuplicates. **(C)** Similar experiments were performed in 4T1 cells treated with verteporfin-PDT and/or 5-ADC (0.5 µM) for 48 h. Concentrations of verteporfin tested at 1 J cm^-2^ and 2.5 J cm^-2^ were 0.25 μM and 0.15 μM respectively. Data are mean +/- SD of three independent experiments performed in sextuplicates. **(D)** Morphological analysis by bright field microscopy of 4T1 cells treated with verteporfin-PDT and/or 5-ADC evaluated 48h post-treatment. **(E)** Western-Blot and immunofluorescence of (cleaved) caspase-3 in 4T1 cells treated with verteporfin-PDT and/or 5-ADC at 1 J.cm^-2^ or 2.5 J.cm^-2^ 48h post-treatment. p<0.05 (*), p<0.01 (**), and p<0.001 (***).

Various *in vitro* assays were used to evaluate the effect of verteporfin-PDT or 5-ADC in monotherapy on the viability and metabolic activity of 4T1 TNBC cells. The IC_50_ of verteporfin was determined at two light doses (1 or 2.5 J cm^-2^) using MTT assays performed 48h after treatment. IC_50_ of verteporfin at 1 J cm^-2^ was between 0.25 and 0.3 μM, while IC_50_ of verteporfin at 2.5 J cm^-2^ was 0.15 μM (**Figure 1A**). The IC_50_ of 5-ADC was achieved at 0.7 μM 48 hours post-treatment (**Figure 1B**).

This was followed by assays to measure the cytotoxic effect of the therapies. Specifically, the ability of 0.5 μM of 5-ADC to enhance the cytotoxic effect of verteporfin-PDT. Therefore, 0.25 μM of verteporfin was used at light dose 1 J cm^-2^ while a concentration of 0.15 μM was investigated at 2.5 J cm-^2^. Treatment of TNBC cells with the combination verteporfin-PDT/5-ADC decreased cell viability to a larger extent than did each treatment alone (p< 0.0001). This effect was observed at both light doses implying that the combination of verteporfin-PDT/5-ADC is significantly more efficient to reduce cell proliferation and viability compared to the monotherapies *in vitro* (**Figures 1C, D**). To determine whether the combination of verteporfin-PDT and 5-ADC exerts a synergistic cytotoxic effect, the synergy was assessed (**Table 2**). Results showed that the combination therapy (verteporfin-PDT/5-ADC) exerted a synergistic cytotoxic effect on 4T1 cells irrespective of the two light doses.

**Table 2:** Synergistic cytotoxic effect of the combination of verteporfin-PDT with 5-ADC on 4T1 TNBC cell line.

Light Dose	[A+B]	[A] x [B]/100	Effect
**1 J cm^-2^**	28.8	32.4	Synergy
**2.5 J cm^-2^**	21.1	27.7	Synergy

[A]: percentage of cell viability after treatment A (verteporfin-PDT 0.15 µM at 1 J.cm^-2^ or 0.25 µM at 1 J.cm^-2^ in monotherapy).

[B] percentage of cell viability after treatment B (0.5 µM 5-ADC in monotherapy), and [A + B] % cell viability after combination treatment (verteporfin-PDT+5-ADC). The effect of the combination treatment is defined as: synergism: [A + B]< [A] x [B]/100; additive: [A + B] = [A] x [B]/100.

*In vitro* experiments were carried out to determine whether the effect of combination therapy (low-light dose PDT with single high dose of 5-ADC) induced cell death by apoptosis. This was achieved by measuring the expression of a key pro-apoptotic protein, cleaved caspase 3, using western blotting and immunofluorescence (**Figure 1E**). Caspases enzymes are crucial mediators of apoptosis. Caspase-3 is a protease that is cleaved and activated during apoptosis to catalyse the cleavage of essential cellular proteins contributing to cell death. It is widely used as a maker of apoptosis and as an indicator of cytotoxicity of potential therapeutic agents (56). Western blot and immunofluorescence of cleaved caspase-3 showed an increase in expression in 4T1 cells with all treatments. All treatments showed increase in apoptosis with the highest observed with combination of verteporfin-PDT/5-ADC at 1 J cm^-2^ with the caspase-3/GAPDH ratio of 10.67, followed by verteporfin-PDT at with ratio of 9.75, at the evaluated timepoint. Interestingly, combination treatment of verteporfin-PDT/5-ADC at 2.5 J cm^-2^ showed lower expression of cleaved caspase-3 than combination treatment at 1 J cm^-2^ with cleaved caspase-3/GAPDH ratio at 4.87 at 48h post-treatment. The reason for this result might be that the PDT treatment at 2.5 J cm^-2^ was more effective than 1 J cm^-2^, and cell death was quickly induced after the end of the illumination period (morphological changes and cleaved caspase-3 expression were already observed 24h post-treatment at 2.5 J cm^-2^; data not shown), and many cells were detached and floating in the wells already 48h post-treatment. Overall, 4T1 cells treated with the combination treatment compared to control untreated cells, at both light doses, showed induction of cell death by apoptosis (**Figure 1E**).

### 3.2 *In vitro combination* therapy of verteporfin-PDT and 5-ADC is more effective than single therapy on tumour suppression via DNA methylation repression and YAP inhibition

DNA methylation is a major epigenetic mechanism that plays an essential role in regulation of gene transcription (35). Aberrant DNA methylation in cancer is involved in tumourigenesis, silencing of tumour suppressor genes, as well as evasion of tumour cells from immune surveillance by suppressing expression of MHC molecules and tumour antigens needed to induce an anti-tumour immune response (36–38). 5-ADC is a methyltransferase inhibitor that inhibits DNA methylation which helps in restoring expression of tumour suppressor genes and tumour antigens, and therefore the initiation of an anti-tumour immunity (39).

**Figure 2:**
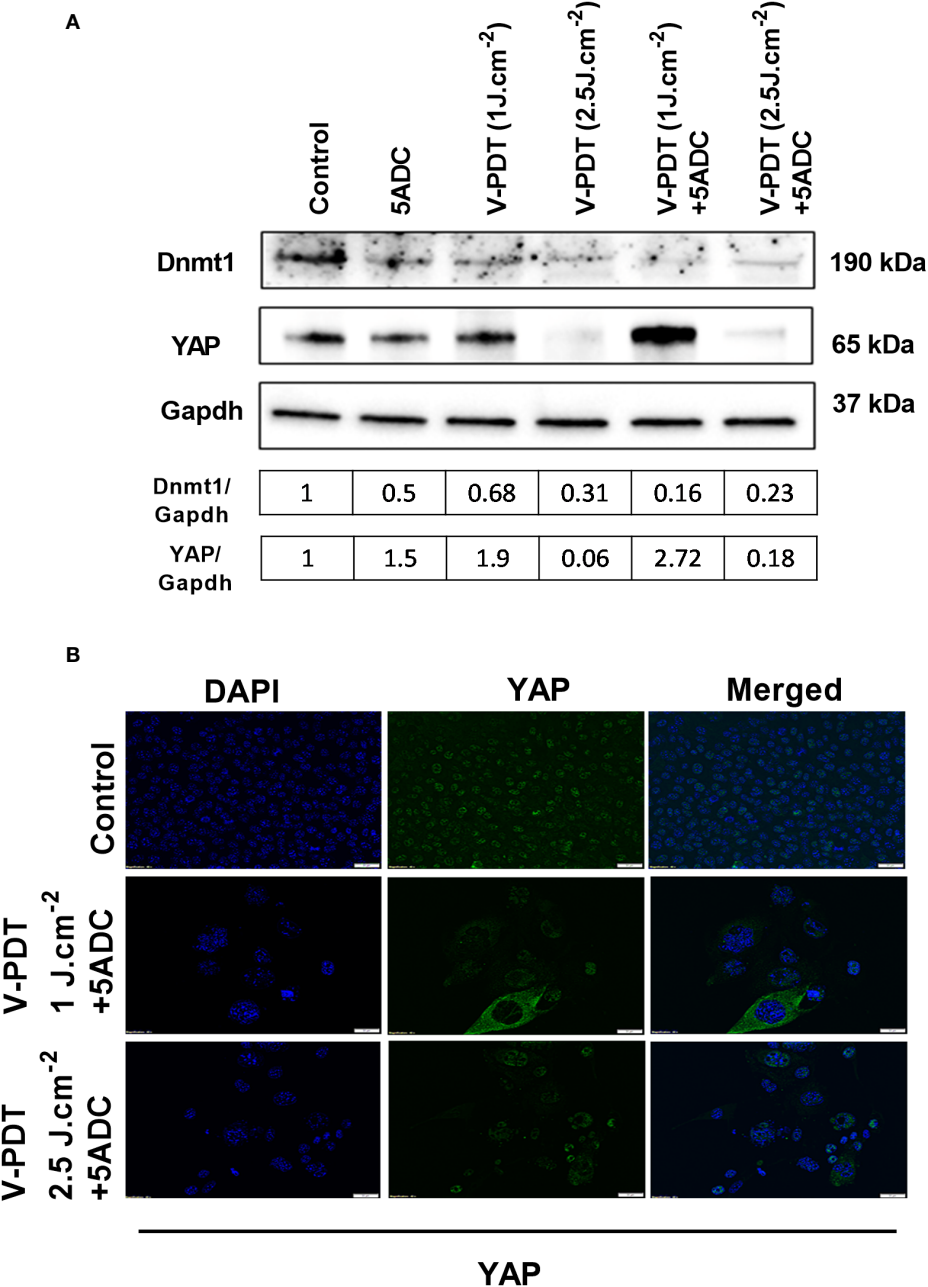
Effects of the combination of verteporfin-PDT with 5-ADC on expression of DNA methylation regulators in 4T1 TNBC cell line. **(A)** Western blot of Dnmt1 and YAP in 4T1 TNBC cells treated with verteporfin-PDT and/or 5-ADC (0.5 µM) evaluated 48 h post-treatment. Concentrations of verteporfin tested at 1 J.cm^-2^ and 2.5 J.cm^-2^ were 0.25 μM and 0.15 μM respectively. **(B)** Immufluorescence staining of YAP in 4T1 cells treated with verteporfin-PDT and/or 5-ADC at 48h post-treatment.

To determine whether the combination therapy affects DNA methylation, the protein expression of two key regulators of DNA methylation, Dnmt1 (DNA methyltransferases 1) and YAP (Yes-associated protein) coded by *YAP1* gene were measured in 4T1 cells treated with verteporfin-PDT with or without 5-ADC. Dnmt1 is the major enzyme responsible of DNA methylation maintenance (35) and defects in Dnmts was shown to cause epigenetic disruption associated with tumourigenesis (57). YAP, one of the major effectors of the Hippo pathway, also contributes to DNA methylation remodeling (58) and its downregulation was shown to have tumour suppressive effects in breast cancer (59). However, *YAP1* was also shown to play a dual role as oncogene and tumour suppressor gene in human oncogenesis depending on the tissue type (60). Several studies have shown that verteporfin can downregulate YAP expression, having an effect on cell proliferation and death (61). Based on this, we wanted to validate a possible synergistic antitumoral effect induced by the double downregulation of Dnmt1 and YAP, promoted by the combination of 5-ADC and verteporfin-PDT. The aim was to prevent cytotoxicity while achieving hypomethylation, immune activation and inhibition of proliferation, migration and invasion by using low-dose exposure of both drugs. The results showed that Dnmt1 was reduced by the mono- and combination treatments, with the highest reduction observed in the combination therapy at both light doses with ratio of Dnmt1/GAPDH at 0.16 for verteporfin-PDT/5-ADC at 1 J cm^-2^ and 0.23 at 2 J cm^-2^, respectively (**Figures 2A, B**). These results suggest that the combination therapy is more efficient in repressing DNA methylation compared to monotherapy at both light doses and therefore could better enhance the anti-tumour immune response.

Regarding YAP, the results showed suppression of its expression at 2.5 J cm^-2^ light dose with both verteporfin-PDT and verteporfin-PDT/5-ADC with YAP/GAPDH ratios of 0.31 and 0.23, respectively, and surprisingly, an increase at 1 J cm^-2^ with YAP/GAPDH ratio of 0.68 for verteporfin-PDT and the highest increase of YAP/GAPDH ratio at 2.72 for verteporfin-PDT/5-ADC treatment (**Figures 2A, B**).

Overall, the *in vitro* experiments demonstrated an improved cytotoxic effect of the combination verteporfin-PDT/5-ADC compared to monotherapies with induction of apoptotic and immune response markers in cancer cells. In addition, the effect of the combination was synergistic at 1 Jcm^-2^ and 2.5 Jcm^-2^ light doses. The low expression of YAP protein at 2.5 Jcm^-2^ light dose indicates that it is more effective on inhibiting breast cancer cells. Based on these results, *in vivo* experiments were conducted in an orthotopic murine breast cancer model to investigate the effect of the combination treatments on tumour growth, cell death as well as anti-tumour immune response.

### 3.3 *In vivo* mono and combination therapy of verteporfin-PDT and 5-ADC show the presence of necrotic areas at the site of PDT treatment in orthotopic murine 4T1 breast cancer model

An appropriate experimental breast cancer model to study the cytotoxic and immunological effects of the combination verteporfin-PDT/5-ADC is the immuno-competent, syngeneic, orthotopic 4T1 breast cancer murine model established in immune-competent Balb/c mice. This experimental model is poorly immunogenic and profoundly immune-suppressive, lacking for instance expression of MHC class II, key regulator of the adaptive immunity (62, 63). In addition, 4T1 has tendency for early metastasis to regional lymph nodes, as well as to distant sites such as the lung, liver, brain and bone (45, 64). These characteristics closely correlate with the pathological progression of high-grade triple receptor negative human breast carcinoma (TNBC) (44) as well as advanced breast cancer which favours its use for *in vivo* investigations (45).

#### 3.3.1 Determination of the optimal timing of PDT delivery after administration of the photosensitizer

The initial set of *in vivo* experiments aimed at determining the optimal timing of PDT delivery after administration of the photosensitizer (PS). Four mice bearing 4T1 breast tumours were given an intravenous injection of verteporfin. PDT was delivered either after 15 minutes or 1 hour after administration of the PS. Each set of mice was either given a light dose of 50 J at 50 mW power setting or 50 J at 100 mW. A higher power could not be used owing to the risk of thermal damage. No adverse events occurred during or after injection of the PS or during anesthesia or PDT. There was no evidence of skin necrosis or any superficial evidence of necrosis of the tumours. The diameters and volumes of the tumour and necrotic area (within the tumour) were measured using histopathological techniques, from which the percentage of necrosis volume versus tumour volume were derived (**Supplementary Figure 1**). Mice treated with PDT 15 minutes after PS injection showed evidence of tumour necrosis at the treatment site. The diameter of necrosis was 4mm and 6mm for 50mW and 100mW, respectively. The extent of this was slightly greater at 100 mw than at 50 mW. In contrast, mice showed no necrosis at the PDT treated site when treated 1 hour after injection of PS at power setting 50mW and 100mW. Although there was 2mm of central patchy necrosis in the tumour treated with 100mW this was not related to the PDT site and resembled central necrosis seen in the control group. This occurred in control tumours 15mm or more in diameter, also a feature commonly seen in rapidly proliferating tumours. The optimum timing for PDT delivery was deemed to be 15 minutes after injection of the PS.

The preliminary results showed that delivery of PDT 15 min after PS is more efficient in reducing tumour volume and inducing tumour necrosis compared to 60 min post-PS, which is consistent with the predominantly vascular localization of the PS within the first 30 minutes of treatment thereby resulting in acute vascular damage. At longer durations post-administration PS would have been taken up by tumour cells, owing to its rapid clearance from circulation thereby reducing the extent of vascular damage.

#### 3.3.2 Determination of the lowest light dose and power setting for consistent necrosis

Determination of the lowest light dose and power setting needed to produce consistent necrosis was carried out next. Two light doses (50J and 90J) were initially considered with power settings of 30mW, 50mW, and 100mW. However, the 30mW power setting was only used for the 50J light dose since 90J would have resulted in a treatment duration of 50 minutes. Nine mice were treated at 3 power settings as follows: 50J at 30mW, 50J and 90J at 50mW, 50J and 90J at 100mW. Results are shown in (**Supplementary Figure 2**). The results demonstrated that 50J at power setting of 50 mW was the lowest light dose and power setting for achieving consistent results. Treatment of tumours with 90J at 100mW showed similar results. The duration of treatment of these energy settings were comparable suggesting that the ‘window’ for optimal treatment duration was no more than about 30 minutes for this treatment group. This is supported by the observations that the least extent of necrosis still visible on microscopy was observed at 50J at 30mW and 90J at 50 mW also resulted in reduced extent of necrosis; both these had prolonged total duration of treatment (inclusive of PS injection) approaching 60 minutes.

#### 3.3.3 Optimum conditions of single and combination therapy of verteporfin-PDT and 5-ADC on orthotopic 4T1 mouse model tumour growth

Following the optimization of the conditions of verteporfin-PDT in terms of power setting, light dose and duration of PDT delivery after verteporfin administration, the effect of the combination verteporfin-PDT/5-ADC on tumour growth in the orthotopic 4T1 breast cancer mouse model was examined. 36 mice were inoculated with 4T1 cells as described above and then divided into 4 groups as follows: Control untreated group (n=10); verteporfin-PDT alone (n=9); 5-ADC alone (6.25mg/Kg) (n=8), or 5-ADC (6.25mg/Kg) followed 48h later with verteporfin-PDT (n=9). In these experiments, verteporfin-PDT was delivered at light dose 50J with 50mW power setting following intravenous PS delivery 15 minutes earlier. Four days after PDT, the diameters of the primary tumours were measured, the volumes calculated, and the necrotic areas were measured manually from histopathology slides by high power microscopy. Local effect on the treated site as well as distant effect and metastases in the spleen were also examined.

**Figure 3:**
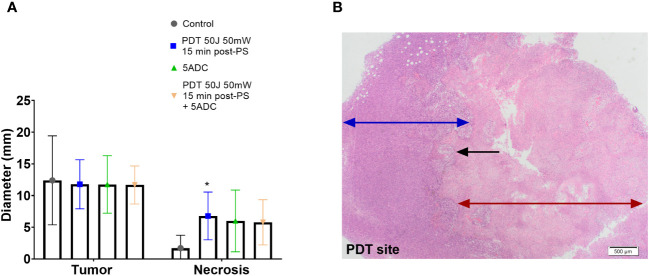
Effect of the combination verteporfin-PDT/5-ADC on tumour growth in an orthotopic murine breast cancer model. Mice were inoculated with 4T1 cells then treated or not with verteporfin-PDT alone (n=9); 5-ADC alone (6.25mg/Kg) (n=8); or 5-ADC (6.25mg/Kg) followed 48h later with verteporfin-PDT (n=9). Verteporfin-PDT was delivered at light dose 50J with 50mW power setting following intravenous PS delivery 15 minutes earlier. **(A)** Tumour and necrotic area diameters were measured using histopathology. **(B)** PDT site with ablated tumour (red arrow), margin with living tumour (blue arrow). The black arrow represents the sharp line of demarcation between these two areas. p<0.05 (*).

Analysis of the tumour and necrosis diameters determined histologically (**Figure 3**) showed variations in the tumour size within each group, while ranges of tumour diameters were similar for the 3 treatment groups. The control group had the widest range of tumour diameters with the largest tumours. Analysis of the tumour volumes showed comparable volumes in the 4 groups. Despite the same volume of tumour cells were implanted, the mice were not immunosuppressed or genetically identical. Therefore, this variation could occur due to differences phenotypic expression of the immune system of the mice. Another explanation may be due to failure of some of the cells to implant in large enough colonies in close vicinity to each other.

Histopathology analysis showed presence of necrosis at the PDT site of mice treated with PDT alone, and mice treated with PDT combined with 5-ADC with no evidence of necrosis at other sites. The median necrotic volume was highest in the PDT alone treatment group (**Figures 3A, B**). Both groups showed a smooth confluent pattern of necrosis radiating circumferentially from the PDT site with a clear margin between necrotic tissue and living tumour cells outside the zone of treatment (**Figure 3B**). There was an intermediate zone of apoptotic cells between these two areas that was more prominently demarcated in the mice receiving combined treatment. However, combining a single high dose 5-ADC prior to PDT did not lead to a greater extent of necrosis at the PDT site although qualitatively the pattern of necrosis at the PDT site was more prominently demarcated in the combined therapy group.

In contrast, mice treated with the 5-ADC alone showed central necrosis characterized by irregular islands of apoptosis and necrotic cells with intervening areas of living tumour cells in most cases, although some had no necrosis. There was no evidence of peripheral necrosis in the tumours of these drug treated mice. The untreated control mice had tumours that did not show necrosis unless the tumour diameter was > 15 mm, a feature commonly seen in rapidly proliferating tumours. The morphology of necrosis assessed by histopathology for each group is summarized in **Table 3**.

**Table 3:** The morphology of necrosis observed in the breast tissue by histopathology.

Group	Morphology pattern/Site of necrosis
Control	No necrosis in most tumours, or patchy irregular islands of central and peripheral necrosis in tumours ≥15mm; no clear demarcation
PDT	Necrosis at PDT site; smooth confluent, highest median necrotic volume.
5-ADC	Patchy islands of central necrosis; no clear demarcation
PDT + 5-ADC	Necrosis at PDT site; smooth confluent; clear demarcation between necrosis and living cells

**Figure 4:**
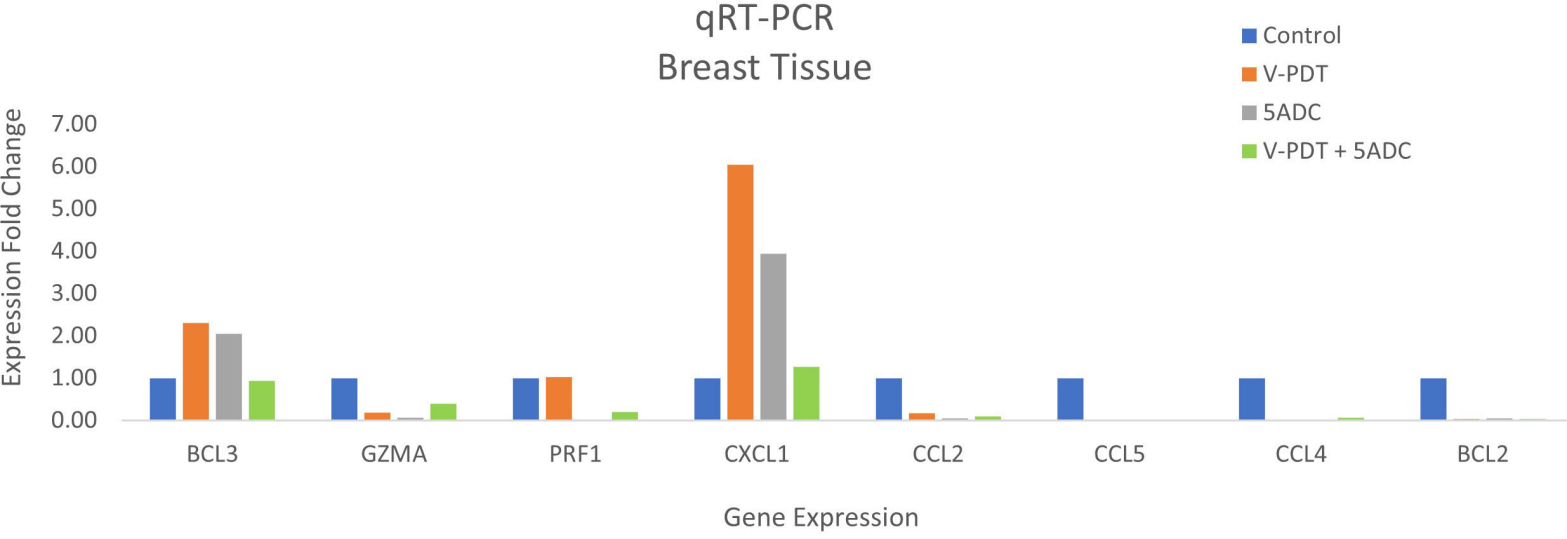
Effects of the combination of verteporfin-PDT with 5-ADC on expression of anti-tumour immune response biomarkers in the breast tissue of murine 4T1 TNBC model.

BCL2 is a pro-survival biomarker, and its overexpression is common in many types of human cancer including breast cancer (65, 66). In TNBC, BCL2 expression was shown to be an independent poor prognostic factor (67). Therefore, we examined levels of the pro-apoptotic marker BCL2 in the breast tissue of treated or untreated mice. Results showed a decrease in BCL2 levels in all treated groups compared to the control indicating apoptosis of tumour cells in the treated groups (**Figure 4**). To summarize, the results showed the presence of necrotic area at the site of PDT treatment delivered as monotherapy or in combination with 5-ADC, although no reduction in tumour size in any of the groups was noted.

### 3.4 *In vivo* mono and combination therapy of verteporfin-PDT and 5-ADC reverses metastasis of cancer cells in orthotopic 4T1 murine breast cancer model

Whether combination therapy could exert suppressing effects on metastasis of tumour cells was examined. Indeed, in addition to its direct cytotoxic effect on malignant cells, PDT is known to induce inflammatory and anti-tumour innate and adaptive immune responses against distant untreated tumour cells allowing the control of distant metastases (15).

The spleen tissue of untreated and treated mice was examined histopathologically to observe the differences in cellular architecture and presence of metastases in the treatment and control groups. Lung, liver and spleen were also examined for the presence of metastases.

All animals injected with the 4T1 cell line displayed metastases in the lungs at six weeks with substantial numbers displaying metastasis to liver (5/6), spleen (3/6) and bone (2/6). Metastases were occasionally found in lymph nodes, brain, intestine, kidneys and adrenals. Splenic metastasis in 4T1 model has been described previously (64).

**Figure 5:**
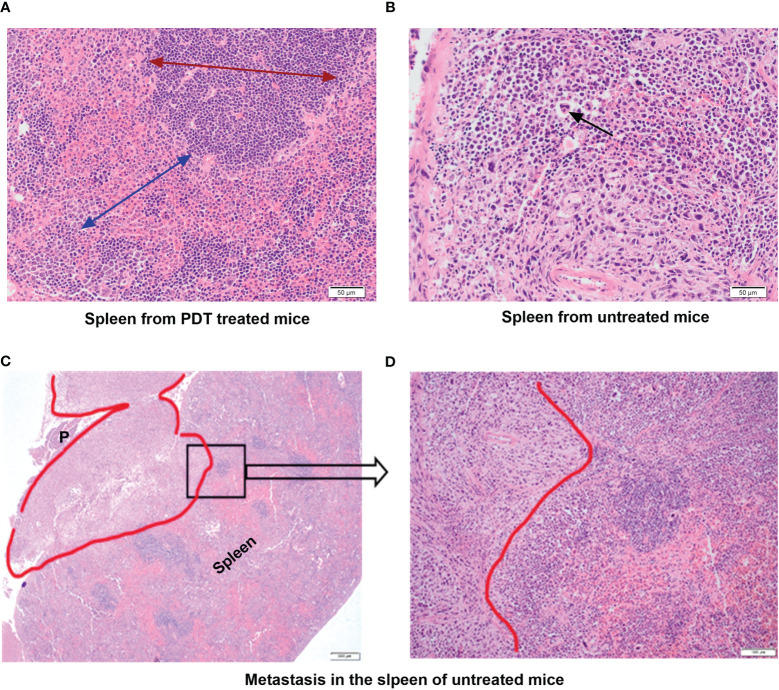
Histopathology assessment of the spleen tissue from mice treated with PDT compared to untreated group. **(A)** Spleen from PDT treated mice showing intact architecture and normal ratio of red and white pulp; the red arrow shows the extent and normal architecture of the red pulp in the spleen; the blue arrow denotes the white pulp region (x40 magnification). **(B)** Spleen from control untreated 4T1 mice showing abnormal splenic architecture and depletion of white pulp with no recognizable regions. The black arrow denotes giant atypical cells found in the inter-follicular medullary region of the spleen (x40 magnification). **(C)** Spleen from control untreated 4T1 mice showing a metastatic tumour deposit (red outline) measuring 7mm. This invades into the peri-splenic soft tissue forming a mass that lies between the spleen and pancreas (P) (x4 magnification). **(D)** High power view of a selected area (black square in C) of the spleen from control untreated 4T1 mice showing the pleomorphic tumour spindle cells invading the adjacent lymphoid tissue of the spleen (x40 magnification).

In the group treated only with PDT there was no evidence of metastases in any of the splenic tissue (**Figure 5A**). In addition, the histopathological appearance of spleens of mice treated with PDT showed that spleens had almost entirely normal architecture with no change in the ratio of red pulp to white pulp and no activated cell expansion (**Figure 5A**). Some specimens did demonstrate expansion of the red pulp, with increased populations of megakaryocytes and atypical cells, but this was much less than seen in the control group mice. In contrast, all control mice showed evidence of infiltration by neutrophils, T lymphocytes and macrophages of splenic tissue with depletion of B cells, and disruption of normal splenic architecture (**Figure 5B**). This included increase in the red pulp with simultaneous reduction in the white pulp and expansion of blast-like cells. The control mice showed evidence of metastatic deposits within the spleen in 20% of mice. Splenic metastasis in the control group is shown in **Figures 5C, D**.

**Figure 6:**
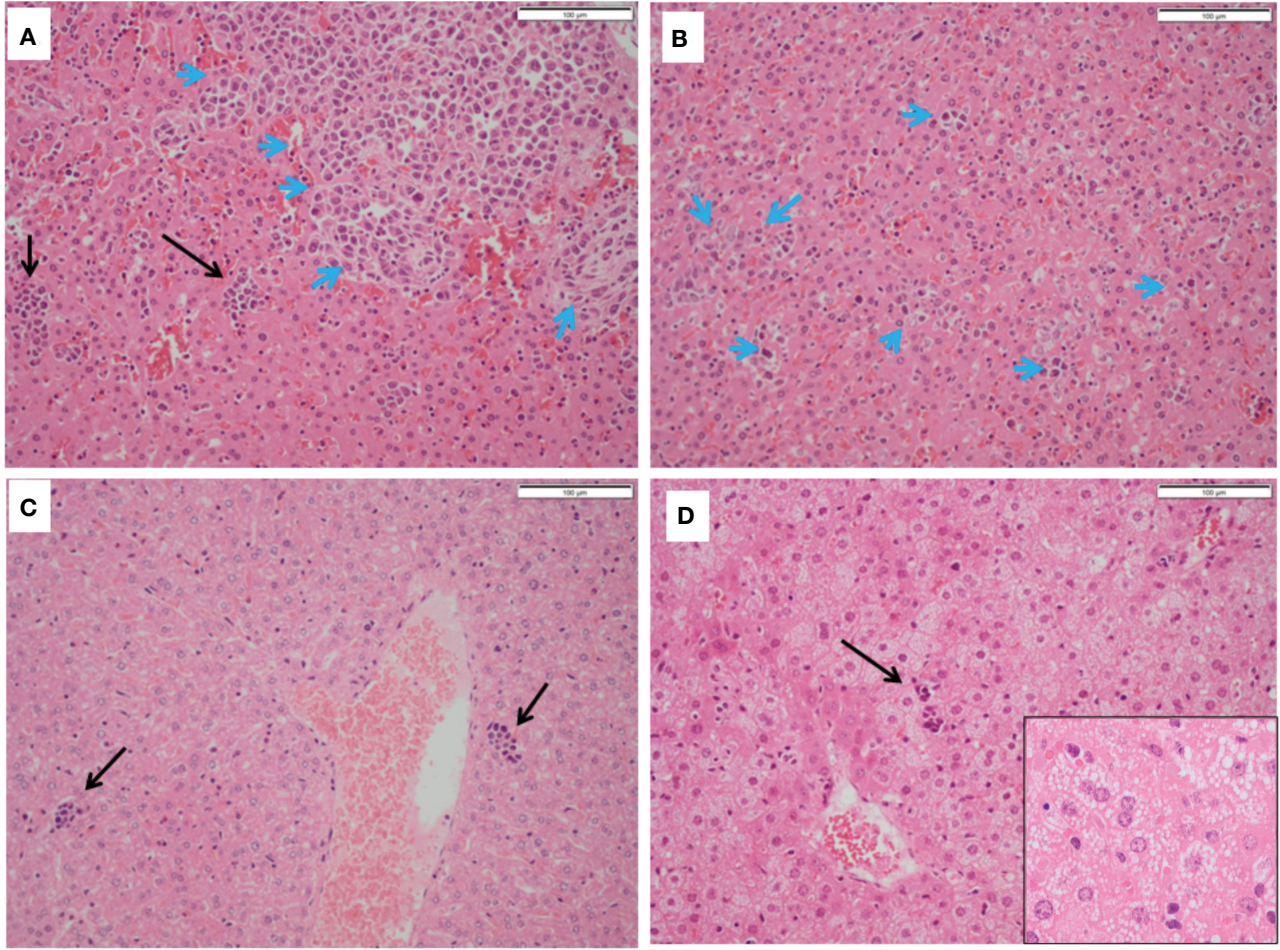
Histopathology of livers from treated and untreated TNBC mice. **(A, B)** Control mice livers containing immune blast forms (blue arrows) with marked infiltration disrupting normal architecture. **(C)** PDT only treated mice livers showing sparse mainly small lymphocyte clusters. **(D)** PDT with 5-ADC treated mice livers showing mild steatosis with sparse small lymphocyte clusters (black arrows).

Mice treated with 5-ADC alone did not show evidence of metastatic deposits in any of the organs sampled but there were increased blast cells and a reduction in white pulp with red pulp expansion in all specimens. This was present in greater proportion than in the mice treated only with PDT, but less than seen in controls. However, unlike control mice there was no disruption of splenic architecture. Mice treated with 5-ADC followed by PDT demonstrated normal splenic tissue with no evidence of metastatic deposits and an intact splenic architecture. The white pulp was preserved with minimal activated immune cell infiltrate in some specimens. The liver specimens from these mice were either entirely normal or showed sparse lymphocytes and blast cell aggregates or mild fatty infiltration of the liver (**Figure 6**). The liver specimens of all the mice treated with PDT alone were entirely normal in architecture with no evidence of metastases.

**Figure 7:**
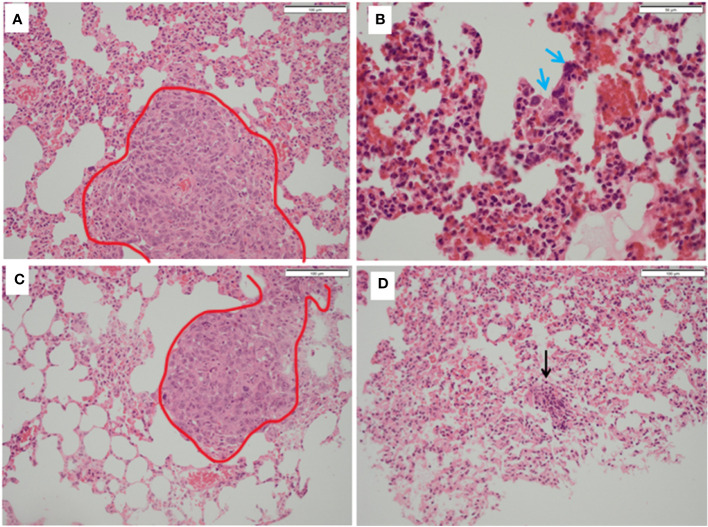
Histopathology of lungs from treated and untreated TNBC mice. **(A)** Control mice lungs showing metastasis (red outline). **(B)** Control mice lungs showing activated lymphocytes (blue arrows). **(C)** PDT only mice lungs showing metastatic deposits in the lung (red outline) which was morphologically identical to that seen in control mice and with no necrosis. **(D)** PDT with 5-ADC lungs were entirely normal or with sparse lymphocytic clusters (black arrow).

In the liver, immune blasts were either present as an aggregate (**Figure 6A** **-** blue arrows) or as interspersed cells that occupied the sinusoids (**Figure 6B** - blue arrows). Livers of mice treated with PDT alone showed sparse groups of small lymphocytes (**Figure 6C**) while those treated with 5-ADC alone or with PDT showing small lymphocyte clusters (**Figure 6D**) and mild hepatocyte steatosis manifested as cytoplasmic micro-vesiculation (**Figure 6D** **inset**). Similarly, in the liver specimens of control mice there was extensive disruption of normal liver architecture with immune cell infiltration throughout. Specifically, lymphoid infiltration was seen as well as blast-like nests with focal infiltration into the liver sinusoids. There was evidence of extralobular and intra-lobular necrosis, with apoptosis of hepatocytes and disintegration of lobules. In addition, peri-portal oedema was also a feature. Two of the ten control mice lungs contained metastatic tumour deposits, the larger was 0.4mm (**Figure 7A**). The remaining specimens showed variable lymphocytic infiltration that included rare single large blast cells (**Figure 7B**). No other abnormality was detected. One of the nine PDT alone-treated mice had a single metastatic deposit in the lung which was morphological identical to that seen in control mice and with no necrosis. The maximum dimension was also 0.4mm (**Figure 7C**). The remaining lung samples taken from 5-ADC alone and verteporfin-PDT/5-ADC treated mice were either entirely normal or contained sparse groups of small lymphocytes (**Figure 7D**). For all the other mice, there were no metastatic deposits or evidence of extensive cellular infiltration in the lungs of control mice in this study. The alveolar structure remained intact with small nests of large activated lymphoid cells resembling primitive blast cells immune cells seen within them. The distribution and frequency of metastasis in the 41T mice is presented in **Supplementary Table 3**.

### 3.5 *In vivo* combination therapy of verteporfin-PDT and 5-ADC result in higher expression of splenic CD4+ and CD8+ T cells in 4T1 breast cancer mice

**Figure 8:**
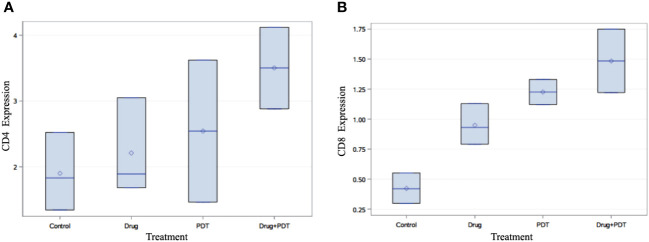
Flow cytometry analysis of splenic tissue of mice treated with verteporfin-PDT and/or 5-ADC. Flow cytometry analysis of **(A)** CD4+ and **(B)** CD8+ T cell populations in each experimental group. The results indicate that PDT treated groups had increase in their population of both CD4+ and CD8+ T cells compared to control mice and those treated 5-ADC alone.

PDT was shown to control distant metastases by possibly inducing anti-tumour immune response. As mentioned above, cell death induced by PDT is accompanied by the release and/or exposure of damage-associated molecular patterns (DAMPs) by the dying cells which causes recruitment of antigen presenting cells (APC) to the site of cellular injury to eliminate photo-damaged tumour cells by phagocytosis. Subsequently, the recruited immune cells present antigen-derived peptides in association with major histocompatibility complex (MHC) molecules to T lymphocytes which results in activation of CD4+ T helper cells, CD8+ cytotoxic T cells as well as B cells and hence the initiation of an adaptive immunity eventually allowing the control of distant metastases (15–22). Thus, the splenic tissue of control and treated mice was examined to determine presence of CD4^+^ and CD8^+^ T-cells using histopathology and flow cytometry analyses. Flow cytometry of splenocytes also showed increased populations of CD4^+^ and CD8^+^ T cell populations in experimental groups in comparison to controls (**Figure 8**).

Flow cytometry analysis, using multivariate analysis with ANOVA followed by Bonferroni *post-hoc* analysis demonstrated significant increase in the expression of CD4+ (p< 0.05) and CD8+ (p< 0.01) in verteporfin-PDT/5-ADC treated mice compared to control (**Figure 8**). In addition, the flow cytometry analysis showed that PDT or 5-ADC only treated groups compared to control had significant increase in CD8+ (p< 0.01) but not CD4+ (p > 0.05). There was no significant difference between the control and verteporfin alone in the CD4+ group. The distribution of distant metastatic deposits in treated and untreated mice is summarized in **Supplementary Table 3**. The overall frequency of metastasis in untreated mice was 4/10 (40%), whereas only 1/26 (4%) mice who received treatment developed metastasis.

### 3.6 Digital pathology analyses show higher expression of splenic CD8+ in all treatment groups and CD4+ with PDT and 5-ADC combination therapy group compared to control in TNBC 4T1 mice

The histopathology of the CD8+ and CD4+ staining was examined using digital pathology analysis of FFPE slides stained with CD4 and CD8 antibodies. The area of the spleen was carefully delineated using the surrounding circumference of the tissue (**Supplementary Figure 3**) and the percentage positivity of the staining was determined using the “IHC profiler” plugin within ImageJ software (**Supplementary Table 2**).

**Figure 9:**
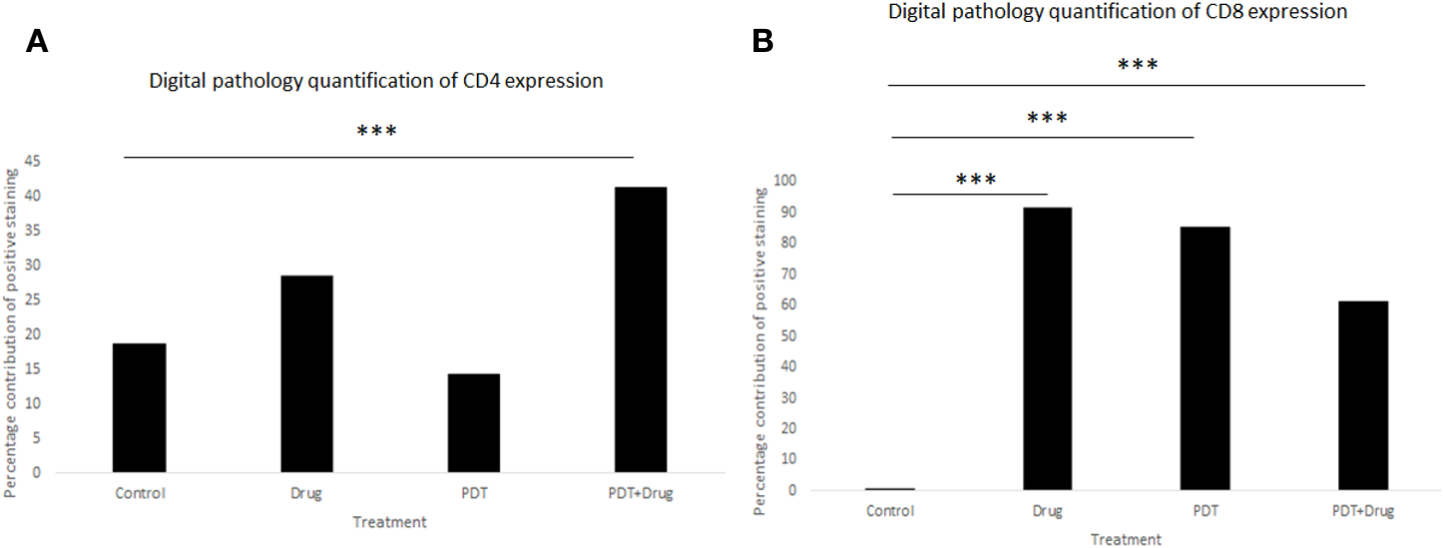
Quantitative percentage of positive staining of **(A)** CD4+ and **(B)** CD8+ using digital pathology. p<0.001 (***).

Digital pathology analysis of CD8+ and CD4+ staining showed that neither CD8+ nor CD4+ had high positivity and that CD8+ had more significant low positivity (p< 0.001) in 5-ADC, verteporfin-PDT and verteporfin-PDT/5-ADC treatment compared to control whereas CD4+ had low positivity (p< 0.001) in the verteporfin-PDT/5-ADC compared to control mice (**Supplementary Table 2** and **Figure 9**). The digital pathology analysis validated the flow cytometry analysis (**Figure 8**) for both the CD4+ and CD8+ groups.

### 3.7 Bioinformatics and *in silico* analysis identifies key anti-tumour immune microenvironment response biomarkers related to TNBC

**Figure 10:**
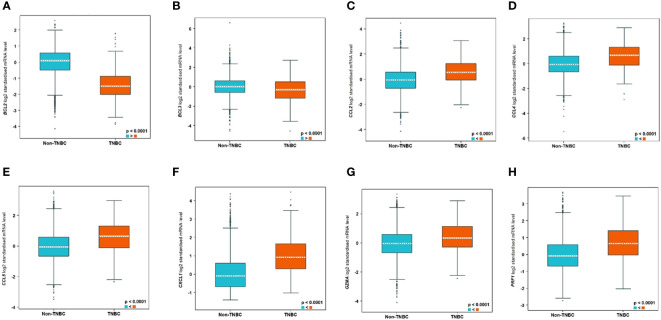
Significantly differentially expressed biomarkers in TNBC compared to non-TNBC subtypes. The differential expression of the genes are as follows: **(A)** BCL2, **(B)** BCL3, **(C)** CCL2, **(D)** CCL4, **(E)** CCL5, **(F)** CXCL1, **(G)** GZMA, and **(H)** PRF1.

Bioinformatics analysis using the bc-GenExMiner tool (http://bcgenex.ico.unicancer.fr) identified 8 tumour immune microenvironment biomarkers related to human TNBC patients. Interestingly, BCL2 and BCL3 showed lower expression in TNBC compared to non-TNBC whereas all the other 6 biomarkers, CCL2, CCL4, CCL5, CXCL1, GZMA, and PRF1, showed higher expression in TNBC (**Figure 10**).

In addition, analysis using the STRING database software showed that the immune response biomarkers form a core connecting the 6 biomarkers with CCL5 being the central hub with BCL2 as well as BCL3 independent of that core, despite the fact that they are differential expressed between TNBC and non-TNBC (**Supplementary Figure 4**).

### 3.8 *In vivo* combination therapy of verteporfin-PDT and 5-ADC on 4T1 orthotopic mouse breast cancer model result in anti-tumour immune response

Investigation of whether combination of PDT with the immunomodulatory agent 5-ADC enhances the anti-tumour immune response compared to monotherapy in mice was carried out by investigating the expression of the anti-tumour immune response genes using qRT-PCR.

Immuno-competent mice were inoculated with 4T1 cells then treated or not with verteporfin-PDT alone (n=9); 5-ADC alone (6.25mg/Kg) (n=8); or 5-ADC (6.25mg/Kg) followed 48h later with verteporfin-PDT (n=9). Verteporfin-PDT was delivered at light dose 50J with 50mW power setting following intravenous PS delivery 15 minutes earlier. Relative gene expression of anti-tumour immune response markers was measured by qRT-PCR. GAPDH was used as internal housekeeping gene. Data are presented as the expression fold change of the mean of three independent experiments performed in duplicates.

**Figure 11:**
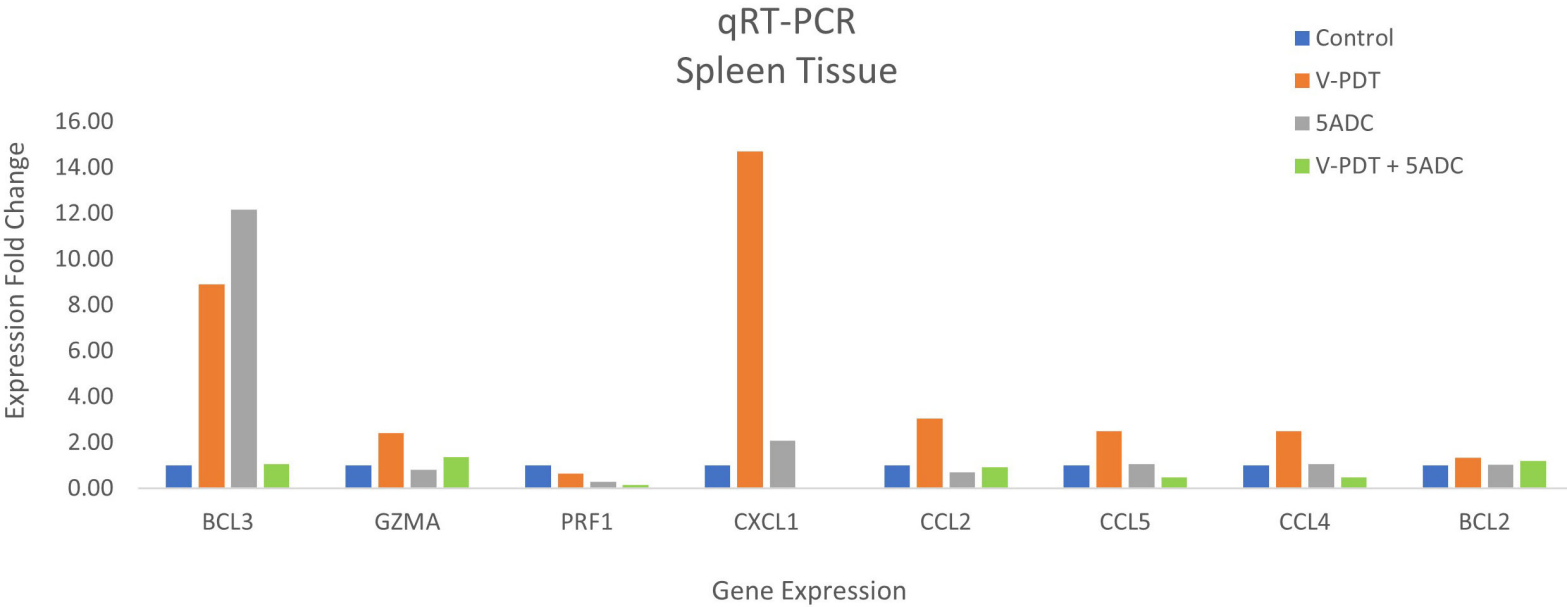
Effects of the combination of verteporfin-PDT and 5-ADC on the expression of anti-tumour immune response biomarkers in the spleen tissue of murine 4T1 TNBC model.

qRT-PCR data from breast tissue show that the NF-kB inhibitor, BCL3, have higher expression in breast tissue from verteporfin-PDT and 5-ADC however they are slightly lower in verteporfin-PDT/5-ADC treated mice compared to control (untreated) mice. All other genes except CXCL1 show decreased expression in treated compared to control (untreated) 4T1 mice (**Table 4** and **Figure 4**). Data from spleen show similar trend to breast between the untreated tumour and treated tissue, however the exception is that with the exception for PRF1 all other genes show higher expression in the verteporfin-PDT with BCL3 and CXCL1 showing the highest similar to that seen in breast tissue. For 5-ADC and verteporfin-PDT/ADC there is decrease but the values are relatively close to the untreated samples (**Table 5** and **Figure 11**).

**Table 4:** Expression fold change of immune response biomarkers from breast tissue of treated compared to control (untreated) TNBC 4T1 mice.

Breast	Control	PDT	5-ADC	PDT/5-ADC
BCL3	1.00	2.31	2.05	0.94
GZMA	1.00	0.18	0.07	0.39
PRF1	1.00	1.02	0.02	0.20
CXCL1	1.00	6.04	3.94	1.27
CCL2	1.00	0.16	0.05	0.09
CCL5	1.00	NA	0.00	0.00
CCL4	1.00	0.02	0.01	0.06
BCL2	1.00	0.03	0.06	0.03

**Table 5:** Expression fold change of immune response biomarkers from spleen tissue of treated compared to control (untreated) TNBC 4T1 mice.

Spleen	Control	PDT	5-ADC	PDT/5-ADC
BCL3	1.00	8.90	12.17	1.06
GZMA	1.00	2.41	0.80	1.36
PRF1	1.00	0.64	0.29	0.16
CXCL1	1.00	14.71	2.08	NA
CCL2	1.00	3.06	0.70	0.91
CCL5	1.00	2.50	1.07	0.49
CCL4	1.00	2.50	1.07	0.49
BCL2	1.00	1.34	1.03	1.19

## 4 Discussion

Photodynamic Therapy (PDT) is currently used for the treatment of various solid tumours. Results from our pilot study of women with breast cancer showed that verteporfin-PDT was effective and well-tolerated (25). There is growing evidence that PDT can elicit an immune response which could enhance its long-term curative efficacy (68). In this experimental study, PDT was assessed *in vitro* and *in vivo* using a 4T1 mouse model in combination with 5-aza-2’-deoxycytidine (5-ADC). Following *in vivo* treatment, tumours were excised, and the extent of necrotic damage was assessed semi-quantitatively using histopathology. Combination treatment of 5-ADC/PDT was assessed to determine if there was synergistic effect present. Apoptotic markers, western blotting techniques and gene expression analysis were used to investigate cell death, immune activation, and cytotoxic effects. For *in vivo* studies mice were sacrificed 5 days after treatment with PDT. Histopathology analysis, digital pathology and immunohistochemistry of treated tumours and distant sites were assessed. Flow cytometry of splenic and breast tissue was used to identify T cell populations. Analysis of breast and splenic tissue to determine the extent of expression of genes associated with immune activation. 5-ADC/PDT therapy showed synergy compared to mono-therapy and was significantly more cytotoxic. The results showed that verteporfin-PDT in combination with 5-ADC exerts a synergistic cytotoxic effect on 4T1 cells irrespective of the dose of light. In addition, *in vivo* treatment with PDT alone and combined therapy demonstrated defined margins of necrosis locally, elevated T cell populations in the spleen with absence of metastases or distant tissue destruction.

As a therapy for human breast cancer, it is likely that verteporfin-PDT would be used in tandem with adjuvant chemotherapy. Thus, the *in vivo* combination therapy of verteporfin-PDT with a cytotoxic agent is a useful translational study. The rationale to use high dose 5-ADC was based on *in vitro* observations of reduction of cell viability at high dose as well as its synergistic effects with verteporfin-PDT presented in this study for the first time in the 4T1 triple negative breast cancer mouse model. Adjuvant chemotherapy typically produces a variable extent of central necrosis with some areas more responsive than others depending on the degree of intra-tumoural heterogeneity present in the cancer. Verteporfin-PDT alone or in combination with 5-ADC caused confluent necrosis at the treatment site with clear margins significantly important to achieving oncological clearance and allowing future comparison with surgical excision. In addition, distant effects observed in the spleen by histopathology and digital pathology correlated with flow cytometry observations of splenocytes following verteporfin-PDT treatment. Furthermore, the increased CD4+ and CD8+ populations in the spleen, observed by flow cytometry and confirmed by digital pathology, correlated with increased BCL3 and reduced BCL2 expression locally suggesting activation of host immune response possibly via the NF-κB pathway. These findings suggest immune-mediated effects in distant organs are controlled at the treatment site demonstrating a link between the histopathology (macro level) and molecular (micro level) events. Suppression of some myeloid cytokines at the treatment site appears to affect distant organs, as no clear evidence of PDT treatment related alteration of gene expression occurred at these sites.

FACS analysis indicated significant incremental increases in CD8 expression along the 5-ADC alone, PDT alone and 5-ADC/PDT sequence compared to untreated mice. Similar analysis indicated increase trend in CD4 expression along the untreated, 5-ADC alone, PDT alone and 5-ADC/PDT sequence. In addition, using digital pathology, CD4 showed significant differences in positivity between 5-ADC/PDT and untreated mice only whereas CD8 showed significant differences across all types of treatments compared to untreated tissue. This clearly shows that adaptive immune response is activated via T cell activation during PDT and 5-ADC/PDT treatments. Functional experiments using the 4T1 cells treated with 5-ADC and in combination of 5-ADC/PDT showed that there is decrease survival as shown by the MTT assays with increase in apoptosis as shown by increase in cleaved caspase-3 expression. Interestingly, there is a decrease in the expression of Dnmt1 with 5-ADC alone, PDT, and 5-ADC/PDT suggesting epigenetic disruption associated with tumourigenesis (33, 57). In addition, the YES-associated protein (YAP), which is a transcriptional coactivator in the Hippo tumour suppressor pathway and is overexpressed in many tumors, showed an increase in expression in PDT and 5-ADC/PDT groups treated with 1 J cm^-2^ but an inhibition of YAP with 2.5 J cm^-2^ at the evaluated time point. The increase in YAP expression observed only when using 1 J cm-2 light dose instead of 2.5 J cm-2 highlights the importance of using optimal PDT treatment parameters to minimise therapeutic resistance. A better inhibition of YAP was achieved by using a lower verteporfin concentration (0.15 μM versus 0.25 μM) and a higher light dose (2.5 J cm-2 versus 1 J cm-2). As previously reported by Wang et al. verteporfin-PDT induced sequestration of YAP in the cytoplasm which is key as nuclear YAP promotes proliferation and EMT potential (**Figures 2A, B**). This can decrease the metastatic potential of 4T1 cells as observed in *in vivo* experiments. This agrees with results by Rashidian et al. showing that YAP inhibition decreased lung metastasis ability in a breast cancer mouse model.

The 4T1 murine model can be difficult to use as an investigative tool for novel treatments, since the rate of tumour growth is unpredictable (47, 69, 70). Because of the aggressive nature of 41T TNBC cells and our observation that control mouse initially died suddenly without significant deterioration 14 days after inoculation, we determined that the biologically relevant response or endpoint relevant to *in vivo* part of the study was the development of acute tumour necrosis. Based on this, the animals were sacrificed 5 days after PDT. At this time, the area of necrosis would still be within the inflammatory phase, before a significant degree of healing by fibrosis would have taken place. A reduction in tumour size was, thus, not expected. Low dose 5-ADC has been associated with increased immune stimulation while high dose produces cytotoxicity in tumours in the clinical setting (32). The results of treatment with repeated low dose 5-ADC in combination with PDT *in vivo* have been modest for 4T1 tumours with 50% regression or prolonged survival compared to controls (28, 31, 71). In other types of tumours, complete regression has been achieved with the same treatment (31, 71). Investigation of adaptive immunity has demonstrated that it may be transferred to untreated mice, then able to resist tumour growth after inoculation. It is not clear if this applied to the 4T1 mouse model (72).

Single high dose application of 5-ADC, used as a point of comparison for the immune-mediated activity of PDT, demonstrated PDT treatment alone exerted comparable if not superior T cell activation to this cytotoxic agent with comparable activation of host immunity and suppression of 4T1 cell activated myeloid cytokines. Combination therapy of PDT with 5-ADC *in vivo* provides circumstantial evidence that there is a possible improved host immune response with combination therapy. BCL3 mediated repression of transcription is associated with the development and activation of immune cells (73). BCL2 on the other hand is recognized as an anti-apoptotic molecule, noted to be upregulated in tumour cells (74). Expression of some genes specific to the 4T1 cell line associated with over-expression of myeloid chemokines, leading to immune-suppression appear to be attenuated by both 5-ADC and PDT and in combination treatment in the breast. Not all genetic biomarkers associated with myeloid over-activity identified by DuPré et al. (50) in the 4T1 TNBC cell line were supressed by PDT, however combination therapy of 5-ADC with PDT suppressed most of the genetic biomarkers associated with myeloid over-activity. This may explain why PDT mono therapy has been less successful in the 4T1 model compared to other *in vivo* models.

Molecular analysis using quantitative real-time PCR (qRT-PCR) for 4T1 mice, showed that the expression of anti-apoptotic BCL2 was attenuated with the NF-kB inhibitor, BCL3, demonstrating increased expression in PDT treated mice indicative of immuno-protective effect. BCL2 had high expression in untreated 4T1 cells but its expression decreased to basal levels with any of the 5-ADC, PDT or 5-ADC/PDT treatments. This indicates that there is probably an increase in apoptosis with the different types of treatments. Interestingly, BCL3 expression show an inverse relationship with BCL2 expression. BCL3 expression in the untreated group showed low expression but increasing with 5-ADC alone and PDT alone indicating the initiation of inflammatory response via NF-κB pathway activation. However, BCL3 expression in 5-ADC with PDT is similar to that in untreated 4T1. Taken together, this could indicate the activation of NF-κB pathway leading to a shift in the downstream transcriptomic profile to increase apoptosis and anti-tumour immune response. This was confirmed by increased expression of different NF-κB inducible immune response genes including GZMA, PRF1, CCL2, CCL4, CCL5, and CXCL1 which also showed similar expression increases in human TNBC patient compared to non-TNBC. Interestingly, the expression of the toxins granzyme A (GZMA) and Perforin 1 (PRF1), secreted by effector cytotoxic T cells and natural killer (NK) cells, were recently shown to be linked to intra-tumoural immune cytolytic activity (75). qRT-PCR results in 4T1 murine breast tissue showed that Granzyme A (GZMA) expression increases gradually along the PDT, 5-ADC, and 5-ADC/PDT sequence indicating T cytotoxic response. However, in untreated cells GZMA expression is higher, probably due to the continuous T cytotoxic response to the tumour cells.

CCL2, also known as monocyte chemoattractant protein 1 (MCP-1), is thought to be an important chemokine for the recruitment of macrophages to the tumour microenvironment (76). In the untreated 4T1 cells, it is higher compared to treated due to the need to respond and contain the tumour as shown by the recent studies linking CD4+ to CCL2 (77). CCL5 (or RANTES) is released by activated T lymphocytes and monocytes/macrophages. CCL2 and its receptor, CCR5, are shown to be involved in cancer cell proliferation, metastasis, and the formation of an immunosuppressive microenvironment (78). The results show a decrease in the level of CCL2 with 5-ADC and 5-ADC with PDT in correlation with tumour depletion. CCL4, also known as macrophage inflammatory protein-1β (MIP-1β), is a chemokine with specificity for CCR5 receptor. It is expressed by natural killer (NK) cells, macrophages, monocytes and a variety of other immune cells. The results show similar response to treatments as that for CCL5.

CXCL1 has a potentially similar role to IL-8. After binding to its receptor CXCR2, CXCL1 activates many pathways implicated in breast cancer including phosphatidylinositol-4,5-bisphosphate 3-kinase-γ (PI3Kγ)/Akt, MAP kinases such as ERK1/ERK2 or phospholipase-β (PLCβ) signaling pathways. CXCL1 is expressed at higher levels during inflammatory responses thus contributing to the process of inflammation (79). CXCL1 has a role in angiogenesis and arteriogenesis and thus has been shown to act in the process of tumour progression (80). Interestingly, the results show large increase in CXCL1 expression with PDT treatment but low expression with the 5-ADC/PDT combination therapy indicating that perhaps with PDT treatment the tumour cells undergo intra-cellular heterogeneity leading to increase in tumour cells, however with combination therapy tumour cell formation is suppressed. In addition, the expression of the anti-tumour response genes in the splenic tissue shows similar pattern to that in the breast tissue however with increase of the expression of the panel of genes in spleen compared to breast tissue. This could be due to the fact that the spleen provides a unique environment combining innate and adaptive immune systems, which can facilitate immediate innate reaction to microbial infection, but also an adaptive immune response that involves the interaction of cells that recognize a particular antigen, implicating MHC molecules presented by antigen-presenting cells. The difference in the breast and spleen environment may explain the differences in the quantitative gene expression values. However, the trend of gene expression is similar with both breast and spleen tissue. Taken together, the study shows that PDT induces anti-tumour immune response with seems to be strongest when treating the TNBC 4T1 mice with combination therapy of PDT with 5-ADC.

The morphological examination using histological data confirmed the molecular data by showing that untreated 4T1 mice exhibited abnormal splenic architecture and depletion of the while pulp, whereas spleen from treated mice show intact architecture with normal ratio of red and white pulp. There was no evidence of metastasis in 25/26 treated mice (one had lung metastasis), even though 40% of control mice showed metastatic TNBC in the lung and liver. This suggest that activation of the immune response via mono therapy with 5-ADC, PDT or 5-ADC/PDT may have activated the immune surveillance mechanism slowing down or preventing metastasis. In addition, PDT enhanced cytotoxic effects of 5-ADC with anti-tumour immune response *in vivo* warrants further investigation on the mechanism involved in the interaction between PDT and 5-ADC on breast cancer cells.

Whilst this study illustrates the importance of immune response with PDT treatment, it has a few limitations. The main limitation is that the study uses 4T1 mice. Having said that, the 4T1 mammary carcinoma has several characteristics that makes it a suitable experimental animal model to study triple negative breast cancer, these include; 4T1 is a transplantable tumor cell line that is highly tumorigenic and invasive and, unlike most other tumor models, can spontaneously metastasize from the primary tumor in the mammary gland to multiple distant sites including lymph nodes, blood, liver, lung, brain, and bone. In addition, we validated the findings of the mouse model with human samples using in silico analysis and showed parallels in the immune response between mouse and human model. Thus, this study ensured that although it might be difficult to find another cell line that have similar properties to 4T1, all the findings have been validated using different techniques and cross referenced with findings from TNBC human patients.

Additional challenges include the fact that studying the effect of combination therapy on immune response, occurs quickly after the end of the PDT treatment and hence many mice were sacrificed at a short-term period (less than 1 week after the end of the treatment). Thus, direct effects on tumour volume, damage to the tumour tissue as well as other parameters could not be evaluated as these require long-term studies. However, due to the aggressive nature of the TNBC model, long-term studies are difficult to avoid metastasis and suffering of control untreated mice or relapse on monotherapy treated mice. Therefore, future research will focus on evaluating changes at the transcriptome level in tumour tissue together with an optimisation of the combinatorial treatment to study if even a better efficacy can be achieved, re-challenging the mice with a second tumour to evaluate immune activation to destroy the tumour cells.

In conclusion, the data from this study constitute the first molecular evidence that PDT is an effective treatment for TNBC. The results also indicate that verteporfin-PDT should be used in sequential treatment with 5-ADC to optimize its efficacy for TNBC therapy. The results also show that some of the immune response genetic biomarkers can be used to monitor the effectiveness of PDT treatment in a TNBC murine model which requires validation in human subjects.

## Data availability statement

The original contributions presented in the study are included in the article/**Supplementary Material**, further inquiries can be directed to the corresponding authors.

## Ethics statement

The animal experiments were undertaken under the ethical approval of the project (UK REC Approval number: 70/7666) and personal licenses (PIL70/25583) granted by UK Home Office (2013) and adhere to the United Kingdom Coordinating Committee of Cancer Research (UKCCCR) guidelines. The study was conducted in accordance with the local rules and institutional requirements.

## Author contributions

SB, MK, RiH and AJM: Conceptualization, data curation, investigation, methodology, writing the original draft preparation, critical review of the manuscript and editing. SB, PA, SS, RaH, NW, AM, GG, AJM and RiH: qRT-PCR, functional *in vitro* and *in vivo* assays and analysis, statistical analysis and data interpretation, writing of the original manuscript, critical review and editing. SB, SS, NW and RiH: samples collection, histopathological diagnosis, IHC interpretation, statistical analysis draft review and editing. RiH: Bioinformatics and digital pathology analysis, writing, critical review, and editing. SB, PA, MK, and AJM: Creating the TNBC murine model, treatment of the TNBC model with PDT, 5-ADC, and combination therapy. All authors contributed to the article and approved the submitted version.

## References

[1] Sung H Ferlay J Siegel RL Laversanne M Soerjomataram I Jemal A. Global Cancer Statistics 2020: GLOBOCAN estimates of incidence and mortality worldwide for 36 cancers in 185 countries. CA Cancer J Clin (2021) 71(3):209–49. doi: 10.3322/caac.21660 33538338

[2] Andre F Pusztai L. Molecular classification of breast cancer: implications for selection of adjuvant chemotherapy. Nat Clin Pract Oncol (2006) 3(11):621–32. doi: 10.1038/ncponc0636 17080180

[3] He J Wang J Li T Chen K Li S Zhang S. SIPL1, regulated by MAZ, promotes tumor progression and predicts poor survival in human triple-negative breast cancer. Front Oncol (2021) 11:766790. doi: 10.3389/fonc.2021.766790 34976812 PMC8718759

[4] Moo TA Sanford R Dang C Morrow M. Overview of breast cancer therapy. PET Clin (2018) 13(3):339–54. doi: 10.1016/j.cpet.2018.02.006 PMC6092031 30100074

[5] Josefsen LB Boyle RW. Photodynamic therapy and the development of metal-based photosensitisers. Metal-based Drugs (2008) 2008:276109. doi: 10.1155/2008/276109 18815617 PMC2535827

[6] MacDonald IJ Dougherty TJ. Basic principles of photodynamic therapy. J Porphyr Phthalocyanines (2001) 5(2):24. doi: 10.1002/jpp.328

[7] Dolmans DE Fukumura D Jain RK. Photodynamic therapy for cancer. Nat Rev Cancer (2003) 3(5):380–7. doi: 10.1038/nrc1071 12724736

[8] Gunaydin G Gedik ME Ayan S. Photodynamic therapy for the treatment and diagnosis of cancer-A review of the current clinical status. Front Chem (2021) 9:686303. doi: 10.3389/fchem.2021.686303 34409014 PMC8365093

[9] van Straten D Mashayekhi V de Bruijn HS Oliveira S Robinson DJ. Oncologic photodynamic therapy: basic principles, current clinical status and future directions. Cancers (Basel) (2017) 9(2). doi: 10.3390/cancers9020019 PMC5332942 28218708

[10] Buytaert E Dewaele M Agostinis P. Molecular effectors of multiple cell death pathways initiated by photodynamic therapy. Biochim Biophys Acta (2007) 1776(1):86–107. doi: 10.1016/j.bbcan.2007.07.001 17693025

[11] Mroz P Yaroslavsky A Kharkwal GB Hamblin MR. Cell death pathways in photodynamic therapy of cancer. Cancers (Basel) (2011) 3(2):2516–39. doi: 10.3390/cancers3022516 PMC3729395 23914299

[12] Oleinick NL Morris RL Belichenko I. The role of apoptosis in response to photodynamic therapy: what, where, why, and how. Photochem Photobiol Sci (2002) 1(1):1–21. doi: 10.1039/b108586g 12659143

[13] Chen B Pogue BW Hoopes PJ Hasan T. Combining vascular and cellular targeting regimens enhances the efficacy of photodynamic therapy. Int J Radiat Oncol Biol Phys (2005) 61(4):1216–26. doi: 10.1016/j.ijrobp.2004.08.006 15752904

[14] Fingar VH Kik PK Haydon PS Cerrito PB Tseng M Abang E. Analysis of acute vascular damage after photodynamic therapy using benzoporphyrin derivative (BPD). Br J Cancer (1999) 79(11-12):1702–8. doi: 10.1038/sj.bjc.6690271 PMC2362794 10206280

[15] Hu Z Rao B Chen S Duanmu J. Targeting tissue factor on tumour cells and angiogenic vascular endothelial cells by factor VII-targeted verteporfin photodynamic therapy for breast cancer in *vitro* and in *vivo* in mice. BMC Cancer (2010) 10:235. doi: 10.1186/1471-2407-10-235 20504328 PMC2882923

[16] Kurohane K Tominaga A Sato K North JR Namba Y Oku N. Photodynamic therapy targeted to tumor-induced angiogenic vessels. Cancer Lett (2001) 167(1):49–56. doi: 10.1016/S0304-3835(01)00475-X 11323098

[17] Reginato E Wolf P Hamblin MR. Immune response after photodynamic therapy increases anti-cancer and anti-bacterial effects. World J Immunol (2014) 4(1):1–11. doi: 10.5411/wji.v4.i1.1 25364655 PMC4214901

[18] Anzengruber F Avci P de Freitas LF Hamblin MR. T-cell mediated anti-tumor immunity after photodynamic therapy: why does it not always work and how can we improve it? Photochem Photobiol Sci (2015) 14(8):1492–509. doi: 10.1039/c4pp00455h PMC4547550 26062987

[19] Castano AP Mroz P Hamblin MR. Photodynamic therapy and anti-tumour immunity. Nat Rev Cancer (2006) 6(7):535–45. doi: 10.1038/nrc1894 PMC2933780 16794636

[20] Kleinovink JW Fransen MF Lowik CW Ossendorp F. Photodynamic-immune checkpoint therapy eradicates local and distant tumors by CD8(+) T cells. Cancer Immunol Res (2017) 5(10):832–8. doi: 10.1158/2326-6066.CIR-17-0055 28851692

[21] Mroz P Hashmi JT Huang YY Lange N Hamblin MR. Stimulation of anti-tumor immunity by photodynamic therapy. Expert Rev Clin Immunol (2011) 7(1):75–91. doi: 10.1586/eci.10.81 21162652 PMC3060712

[22] Mroz P Szokalska A Wu MX Hamblin MR. Photodynamic therapy of tumors can lead to development of systemic antigen-specific immune response. PloS One (2010) 5(12):e15194. doi: 10.1371/journal.pone.0015194 21179470 PMC3001867

[23] Thong PS Ong KW Goh NS Kho KW Manivasager V Bhuvaneswari R. Photodynamic-therapy-activated immune response against distant untreated tumours in recurrent angiosarcoma. Lancet Oncol (2007) 8(10):950–2. doi: 10.1016/S1470-2045(07)70318-2 17913664

[24] Wachowska M Muchowicz A Demkow U. Immunological aspects of antitumor photodynamic therapy outcome. Cent Eur J Immunol (2015) 40(4):481–5. doi: 10.5114/ceji.2015.56974 PMC4737746 26862314

[25] Banerjee SM El-Sheikh S Malhotra A Mosse CA Parker S Williams NR. Photodynamic therapy in primary breast cancer. J Clin Med (2020) 9(2). doi: 10.3390/jcm9020483 PMC7074474 32050675

[26] Shams M Owczarczak B Manderscheid-Kern P Bellnier DA Gollnick SO. Development of photodynamic therapy regimens that control primary tumor growth and inhibit secondary disease. Cancer immunol immunother CII (2015) 64(3):287–97. doi: 10.1007/s00262-014-1633-9 PMC4341021 25384911

[27] Shen Z Ma Q Zhou X Zhang G Hao G Sun Y. Strategies to improve photodynamic therapy efficacy by relieving the tumor hypoxia environment. NPG Asia Mater (2021) 13(39). doi: 10.1038/s41427-021-00303-1

[28] Xia Y Gupta GK Castano AP Mroz P Avci P Hamblin MR. CpG oligodeoxynucleotide as immune adjuvant enhances photodynamic therapy response in murine metastatic breast cancer. J Biophotonics (2014) 7(11-12):897–905. doi: 10.1002/jbio.201300072 23922221 PMC3917974

[29] Korbelik M Sun J Posakony JJ. Interaction between photodynamic therapy and BCG immunotherapy responsible for the reduced recurrence of treated mouse tumors. Photochem Photobiol (2001) 73(4):403–9. doi: 10.1562/0031-8655(2001)073<0403:IBPTAB>2.0.CO;2 11332036

[30] Mohammadpour H Majidzadeh AK. Antitumor effect of conditioned media derived from murine MSCs and 5-aminolevulinic acid (5-ALA) mediated photodynamic therapy in breast cancer in vitro. Photodiagnosis Photodyn Ther (2015) 12(2):238–43. doi: 10.1016/j.pdpdt.2015.02.004 25721458

[31] Wachowska M Gabrysiak M Muchowicz A Bednarek W Barankiewicz J Rygiel T. 5-Aza-2'-deoxycytidine potentiates antitumour immune response induced by photodynamic therapy. Eur J Cancer (2014) 50(7):1370–81. doi: 10.1016/j.ejca.2014.01.017 PMC4136636 24559534

[32] Karahoca M Momparler RL. Pharmacokinetic and pharmacodynamic analysis of 5-aza-2'-deoxycytidine (decitabine) in the design of its dose-schedule for cancer therapy. Clin Epigenet (2013) 5(1):3. doi: 10.1186/1868-7083-5-3 PMC3570332 23369223

[33] Nguyen J Coyne GHO Takebe N Naqash AR Mukherjee J Bruns A. Phase I trial of 5-aza-4’-thio-2’-deoxycytidine (Aza-TdC) in patients with advanced solid tumors. J Clin Oncol (2021) 39(15_suppl):3088–. doi: 10.1200/JCO.2021.39.15_suppl.3088

[34] Moore LD Le T Fan G. DNA methylation and its basic function. Neuropsychopharmacology (2013) 38(1):23–38. doi: 10.1038/npp.2012.112 22781841 PMC3521964

[35] Campoy EM Branham MT Mayorga LS Roque M. Intratumor heterogeneity index of breast carcinomas based on DNA methylation profiles. BMC Cancer (2019) 19(1):328. 30953488 10.1186/s12885-019-5550-3 PMC6451266

[36] Das PM Singal R. DNA methylation and cancer. J Clin Oncol (2004) 22(22):4632–42. doi: 10.1200/JCO.2004.07.151 15542813

[37] Jung H Kim HS Kim JY Sun JM Ahn JS Ahn MJ. DNA methylation loss promotes immune evasion of tumours with high mutation and copy number load. Nat Commun (2019) 10(1):4278. doi: 10.1038/s41467-019-12159-9 31537801 PMC6753140

[38] Klar AS Gopinadh J Kleber S Wadle A Renner C. Treatment with 5-aza-2'-deoxycytidine induces expression of NY-ESO-1 and facilitates cytotoxic T lymphocyte-mediated tumor cell killing. PloS One (2015) 10(10):e0139221. doi: 10.1371/journal.pone.0139221 26447882 PMC4598131

[39] Bown SG Rogowska AZ Whitelaw DE Lees WR Lovat LB Ripley P. Photodynamic therapy for cancer of the pancreas. Gut (2002) 50(4):549–57. doi: 10.1136/gut.50.4.549 PMC1773165 11889078

[40] Sultan AA Jerjes W Berg K Hogset A Mosse CA Hamoudi R. Disulfonated tetraphenyl chlorin (TPCS2a)-induced photochemical internalisation of bleomycin in patients with solid Malignancies: a phase 1, dose-escalation, first-in-man trial. Lancet Oncol (2016) 17(9):1217–29. doi: 10.1016/S1470-2045(16)30224-8 27475428

[41] Huggett MT Jermyn M Gillams A Illing R Mosse S Novelli M. Phase I/II study of verteporfin photodynamic therapy in locally advanced pancreatic cancer. Br J Cancer (2014) 110(7):1698–704. doi: 10.1038/bjc.2014.95 PMC3974098 24569464

[42] Ichikawa K Takeuchi Y Yonezawa S Hikita T Kurohane K Namba Y. Antiangiogenic photodynamic therapy (PDT) using Visudyne causes effective suppression of tumor growth. Cancer Lett (2004) 205(1):39–48. doi: 10.1016/j.canlet.2003.10.001 15036659

[43] Kaplanov I Carmi Y Kornetsky R Shemesh A Shurin GV Shurin MR. Blocking IL-1beta reverses the immunosuppression in mouse breast cancer and synergizes with anti-PD-1 for tumor abrogation. Proc Natl Acad Sci USA (2019) 116(4):1361–9. doi: 10.1073/pnas.1812266115 PMC6347724 30545915

[44] Pulaski BA Ostrand-Rosenberg S. Mouse 4T1 breast tumor model. Curr Protoc Immunol (2001) 20(20.2). doi: 10.1002/0471142735.im2002s39 18432775

[45] Heppner GH Miller FR Shekhar PM. Nontransgenic models of breast cancer. Breast Cancer Res (2000) 2(5):331–4. doi: 10.1186/bcr77 PMC138654 11250725

[46] Kaur P Nagaraja GM Zheng H Gizachew D Galukande M Krishnan S. A mouse model for triple-negative breast cancer tumor-initiating cells (TNBC-TICs) exhibits similar aggressive phenotype to the human disease. BMC Cancer (2012) 12:120. doi: 10.1186/1471-2407-12-120 22452810 PMC3340297

[47] Jezequel P Gouraud W Ben Azzouz F Guerin-Charbonnel C Juin PP Lasla H. bc-GenExMiner 4.5: new mining module computes breast cancer differential gene expression analyses. Database (Oxford) (2021) 2021. doi: 10.1093/database/baab007 PMC7904047 33599248

[48] Szklarczyk D Gable AL Nastou KC Lyon D Kirsch R Pyysalo S. The STRING database in 2021: customizable protein-protein networks, and functional characterization of user-uploaded gene/measurement sets. Nucleic Acids Res (2021) 49(D1):D605–12. doi: 10.1093/nar/gkaa1074 PMC7779004 33237311

[49] DuPré SA Redelman D Hunter KW Jr. The mouse mammary carcinoma 4T1: characterization of the cellular landscape of primary tumours and metastatic tumour foci. Int J Exp Pathol (2007) 88(5):351–60. doi: 10.1111/j.1365-2613.2007.00539.x PMC2517332 17877537

[50] Livak KJ Schmittgen TD. Analysis of relative gene expression data using real-time quantitative PCR and the 2(-Delta Delta C(T)) Method. Methods (2001) 25(4):402–8. doi: 10.1006/meth.2001.1262 11846609

[51] Berryman Z Bridger L Hussaini HM Rich AM Atieh M Tawse-Smith A. Titanium particles: An emerging risk factor for peri-implant bone loss. Saudi Dental J (2020) 32(6):283–92. doi: 10.1016/j.sdentj.2019.09.008 PMC7452065 32874068

[52] Acedo P Stockert JC Canete M Villanueva A. Two combined photosensitizers: a goal for more effective photodynamic therapy of cancer. Cell Death Dis (2014) 5(3):e1122. doi: 10.1038/cddis.2014.77 24625981 PMC3973236

[53] Valeriote F Lin H. Synergistic interaction of anticancer agents: a cellular perspective. Cancer Chemother Rep (1975) 59(5):895–900. 1203896

[54] Martinez de Pinillos Bayona A Woodhams JH Pye H Hamoudi RA Moore CM MacRobert AJ. Efficacy of photochemical internalisation using disulfonated chlorin and porphyrin photosensitisers: An in *vitro* study in 2D and 3D prostate cancer models. Cancer Lett (2017) 393:68–75. doi: 10.1016/j.canlet.2017.02.018 28223166 PMC5360193

[55] Porter AG Janicke RU. Emerging roles of caspase-3 in apoptosis. Cell Death Differ (1999) 6(2):99–104. doi: 10.1038/sj.cdd.4400476 10200555

[56] Kagan AB Garrison DA Anders NM Webster JA Baker SD Yegnasubramanian S. DNA methyltransferase inhibitor exposure-response: challenges and opportunities. Clin Transl Sci (2023) 16(8):1309–22. 10.1111/cts.13548 PMC10432879 37345219

[57] Zhang W Xu J. DNA methyltransferases and their roles in tumorigenesis. biomark Res (2017) 5:1. doi: 10.1186/s40364-017-0081-z 28127428 PMC5251331

[58] Passaro F De Martino I Zambelli F Di Benedetto G Barbato M D'Erchia AM. YAP contributes to DNA methylation remodeling upon mouse embryonic stem cell differentiation. J Biol Chem (2021) 296:100138. doi: 10.1074/jbc.RA120.015896 33268382 PMC7948423

[59] Real SAS Parveen F Rehman AU Khan MA Deo SVS Shukla NK. Aberrant promoter methylation of YAP gene and its subsequent downregulation in Indian breast cancer patients. BMC Cancer (2018) 18(1):711. doi: 10.1186/s12885-018-4627-8 29970036 PMC6031145

[60] Wang H Du YC Zhou XJ Liu H Tang SC. The dual functions of YAP-1 to promote and inhibit cell growth in human Malignancy. Cancer Metastasis Rev (2014) 33(1):173–81. doi: 10.1007/s10555-013-9463-3 24346160

[61] Wang C Zhu X Feng W Yu Y Jeong K Guo W. Verteporfin inhibits YAP function through up-regulating 14-3-3σ sequestering YAP in the cytoplasm. Am J Cancer Res (2016) 6(1):27–37. 27073720 PMC4759394

[62] Schrors B Boegel S Albrecht C Bukur T Bukur V Holtstrater C. Multi-omics characterization of the 4T1 murine mammary gland tumor model. Front Oncol (2020) 10:1195. doi: 10.3389/fonc.2020.01195 32793490 PMC7390911

[63] Wieczorek M Abualrous ET Sticht J Alvaro-Benito M Stolzenberg S Noe F. Major histocompatibility complex (MHC) class I and MHC class II proteins: conformational plasticity in antigen presentation. Front Immunol (2017) 8:292. doi: 10.3389/fimmu.2017.00292 28367149 PMC5355494

[64] Pulaski BA Ostrand-Rosenberg S. Reduction of established spontaneous mammary carcinoma metastases following immunotherapy with major histocompatibility complex class II and B71 cell-based tumor vaccines. Cancer Res (1998) 58(7):1486–93. 9537252

[65] Czabotar PE Lessene G Strasser A Adams JM. Control of apoptosis by the BCL-2 protein family: implications for physiology and therapy. Nat Rev Mol Cell Biol (2014) 15(1):49–63. doi: 10.1038/nrm3722 24355989

[66] Yip KW Reed JC. Bcl-2 family proteins and cancer. Oncogene (2008) 27(50):6398–406. doi: 10.1038/onc.2008.307 18955968

[67] Honma N Horii R Ito Y Saji S Younes M Iwase T. Differences in clinical importance of Bcl-2 in breast cancer according to hormone receptors status or adjuvant endocrine therapy. BMC Cancer (2015) 15:698. doi: 10.1186/s12885-015-1686-y 26472348 PMC4607008

[68] Huang K Yan M Zhang H Xue J Chen J. A phthalocyanine-based photosensitizer for effectively combating triple negative breast cancer with enhanced photodynamic anticancer activity and immune response. Eur J Med Chem (2022) 241:114644. doi: 10.1016/j.ejmech.2022.114644 35939997

[69] Chen L Huang TG Meseck M Mandeli J Fallon J Woo SL. Rejection of metastatic 4T1 breast cancer by attenuation of Treg cells in combination with immune stimulation. Mol Ther J Am Soc Gene Ther (2007) 15(12):2194–202. doi: 10.1038/sj.mt.6300310 17968355

[70] Tao K Fang M Alroy J Sahagian GG. Imagable 4T1 model for the study of late stage breast cancer. BMC Cancer (2008) 8:228. doi: 10.1186/1471-2407-8-228 18691423 PMC2529338

[71] Wachowska M Gabrysiak M Golab J. Epigenetic remodeling combined with photodynamic therapy elicits anticancer immune responses. Oncoimmunology (2014) 3:e28837. doi: 10.4161/onci.28837 25057447 PMC4091536

[72] Almeida RD Manadas BJ Carvalho AP Duarte CB. Intracellular signaling mechanisms in photodynamic therapy. Biochim Biophys Acta (2004) 1704(2):59–86. doi: 10.1016/j.bbcan.2004.05.003 15363861

[73] Wessells J Baer M Young HA Claudio E Brown K Siebenlist U. BCL-3 and NF-kappaB p50 attenuate lipopolysaccharide-induced inflammatory responses in macrophages. J Biol Chem (2004) 279(48):49995–50003. doi: 10.1074/jbc.M404246200 15465827

[74] Vaux DL Cory S Adams JM. Bcl-2 gene promotes haemopoietic cell survival and cooperates with c-myc to immortalize pre-B cells. Nature (1988) 335(6189):440–2. doi: 10.1038/335440a0 3262202

[75] Hu Q Nonaka K Wakiyama H Miyashita Y Fujimoto Y Jogo T. Cytolytic activity score as a biomarker for antitumor immunity and clinical outcome in patients with gastric cancer. Cancer Med (2021) 10(9):3129–38. doi: 10.1002/cam4.3828 PMC8085935 33769705

[76] Jin J Lin J Xu A Lou J Qian C Li X. CCL2: an important mediator between tumor cells and host cells in tumor microenvironment. Front Oncol (2021) 11:722916. doi: 10.3389/fonc.2021.722916 34386431 PMC8354025

[77] Hao Q Vadgama JV Wang P. CCL2/CCR2 signaling in cancer pathogenesis. Cell Commun Signal (2020) 18(1):82. doi: 10.1186/s12964-020-00589-8 32471499 PMC7257158

[78] Li M Knight DA Sydner LA Smyth MJ Stewart TJ. A role for CCL2 in both tumor progression and immunosurveillance. Oncoimmunology (2013) 2(7):e25474. doi: 10.4161/onci.25474 24073384 PMC3782157

[79] Silva RL Lopes AH Guimaraes RM Cunha TM. CXCL1/CXCR2 signaling in pathological pain: Role in peripheral and central sensitization. Neurobiol Dis (2017) 105:109–16. doi: 10.1016/j.nbd.2017.06.001 28587921

[80] Vries MH Wagenaar A Verbruggen SE Molin DG Dijkgraaf I Hackeng TH. CXCL1 promotes arteriogenesis through enhanced monocyte recruitment into the peri-collateral space. Angiogenesis (2015) 18(2):163–71. doi: 10.1007/s10456-014-9454-1 25490937

