# Supplementary material for: Combination of verteporfin-photodynamic therapy with 5-aza-2’-deoxycytidine enhances the anti-tumour immune response in triple negative breast cancer
Source: Front Immunol. 2023 Nov 7;14:1188087. doi: 10.3389/fimmu.2023.1188087 (PMC10664979; doi:10.3389/fimmu.2023.1188087)
Supplement: Supplementary file 1 [file DataSheet_1.docx]

Combination of verteporfin-photodynamic therapy with 5-aza-2’-deoxycytidine enhances the anti-tumour immune response in triple negative breast cancer

**Shramana M. Banerjee^1,2,†^, Pilar Acedo^2,3,†^, Soha El Sheikh^4^, Rania Harati^5^, Amelia Meecham^4^, Norman R. Williams^2^, Gareth Gerard^4^, Mohammed R.S. Keshtgar^1,2^, Alexander J. MacRobert^2,^*, Rifat Hamoudi^2,6,^***

^1^Breast Unit, Royal Free London NHS Foundation Trust, London, United Kingdom

^2^Division of Surgery and Interventional Science, University College London, London, United Kingdom

^3^Institute for Liver and Digestive Health, Division of Medicine, University College London, London, United Kingdom

^4^UCL Cancer Institute, University College London, London, United Kingdom

^5^Department of Pharmacy Practice and Pharmacotherapeutics, College of Pharmacy, University of Sharjah, United Arab Emirates

^6^Research Institute for Medical and Health Sciences, College of Medicine, University of Sharjah, United Arab Emirates

**^†^** These authors have contributed equally to this work.

*** Corresponding Authors:**

A.J. MacRobert: [a.macrobert@ucl.ac.uk](mailto:a.macrobert@ucl.ac.uk)
R. Hamoudi: [rhamoudi@sharjah.ac.ae](mailto:rhamoudi@sharjah.ac.ae) and [r.hamoudi@ucl.ac.uk](mailto:r.hamoudi@ucl.ac.uk)

**Keywords:** Verteporfin, Photodynamic Therapy, 5-Aza-Deoxycytidine, Anti-tumour immune response, Triple Negative Breast Cancer, 4T1

1. **Supplementary Material**


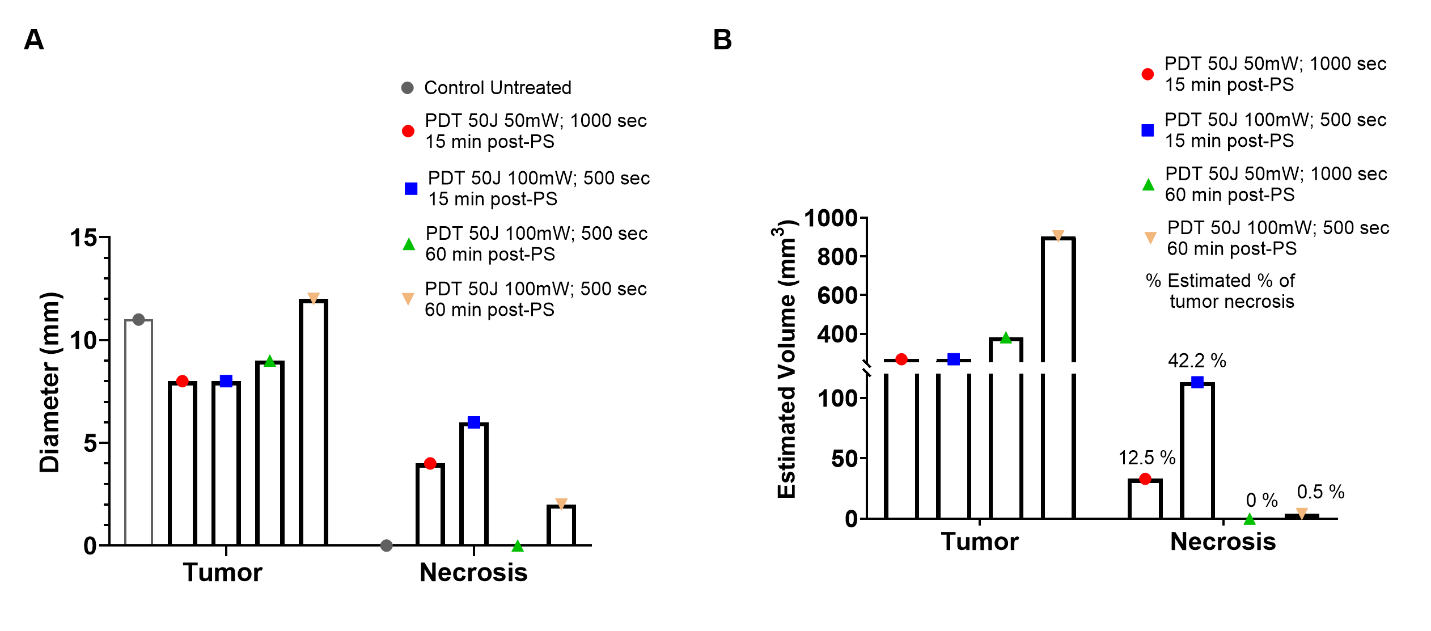


**Supplementary Figure 1.** Optimization of PDT delivery timing. Four mice bearing 4T1 breast tumours were given an intravenous injection of verteporfin and PDT was delivered 15 or 60 minutes after administration of the PS. Each mouse was either given a light dose of 50 J at 50 mW or 50 J at 100 mW. (A) Tumour diameters measured using histopathology techniques (B) Estimated volume was calculated by formula 4/3 πr^3^ to the nearest mm^3^. Necrotic areas (within the tumour) were measured by histopathology, from which the percentage of necrosis volume versus tumour volume was derived.


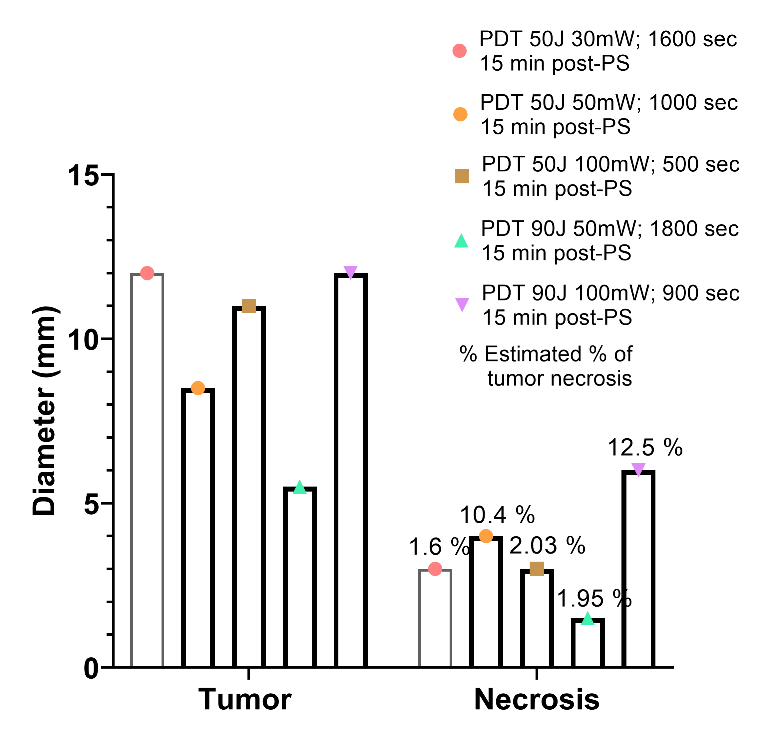


**Supplementary Figure 2.** Optimization of PDT power setting and light dose. Nine mice bearing 4T1 breast tumours were treated at 3 power settings: 50J at 30mW, 50J and 90J at 50mW, 50J and 90J at 100mW. Tumour and necrosis diameters were measured by histopathology..


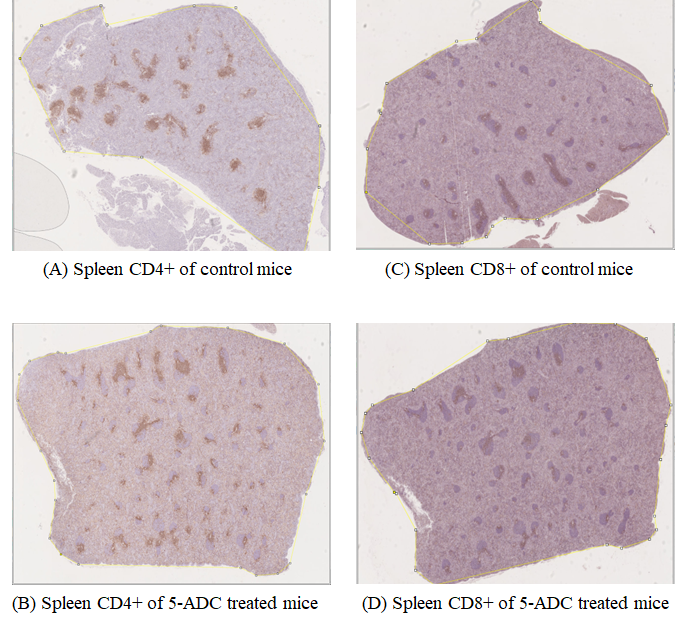


**Supplementary Figure 3.** Delineation of the spleen stained with CD4 and CD8 control and treated with 5-ADC.


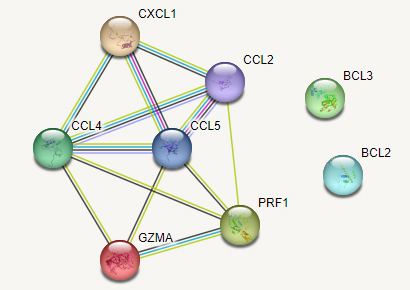


**Supplementary Figure 4.** Graph theoretic protein-protein interaction network of the immune response biomarkers using STRING. The 8 biomarkers are: BCL2, BCL3, CCL2, CCL4, CCL5, CXCL1, GZMA, PRF1

**Supplementary Table 1: Forward and reverse sequences of the primers used in quantitative real-time PCR**

| **Gene** | **Forward Sequence** | **Reverse Sequence** |
| --- | --- | --- |
| BCL3 | CCTTTGATGCCCATTTACTCTA | AGCGGCTATGTTATTCTGGAC |
| GZMA | TTTCTGGCATCCTCTCTCTCA | GGGTCATAGCATGGATAGGG |
| PRF1 | CAGTACAGCTTCAGCACTGAC | ATGAAGTGGGTGCCGTAGTTG |
| CXCL1 | GCTGGCTTCTGACAACACTATA | TGAACAAGCAGAACTGAACTACC |
| CCL2 | AGGTGTCCCAAAGAAGCTGT | TGCTTGAGGTGGTTGTGGAA |
| CCL5 | AAGAAGTTCAGCTGCCCCAT | CGCTCAGCTTTCCTATTACCAA |
| CCL4 | TCCTGCTGTTTCTCTTACACCT | ATGCAGGTGGCAGGAATGTT |
| CXCR4 | AGCATGACGGACAAGTACAGG | GATGAAGTCGGGAATAGTCAGC |
| LAMP1 | ATGGCCAGCTTCTCTGCCTCC | ACAGTGGGGTTTGTGGGCAC |
| BCL2 | CTGCACCTGACGCCCTTCACC | CACATGACCCCACCGAACTCAAAGA |

**Supplementary Table 2**. Digital pathology percentage positivity scoring for splenic CD4+ and CD8+ T-cells in 4T1 mice using the IHC profiler plugin


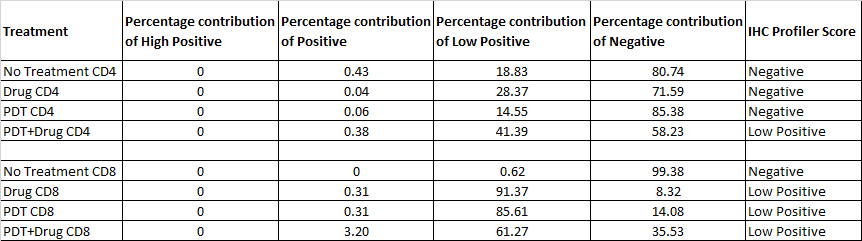


**Supplementary Table 3.** Distribution and frequency of metastasis in the 4T1 mice

| Group | Number of mice affected | Spleen | Lung | Liver |
| --- | --- | --- | --- | --- |
| Control | 4/10 | 2 | 2 | 0 |
| V-PDT | 1/9 | 0 | 1 | 0 |
| 5-ADC | 0/8 | 0 | 0 | 0 |
| V-PDT + 5-ADC | 0/9 | 0 | 0 | 0 |
| Total | 5/36 | 2 | 3 | 0 |

**
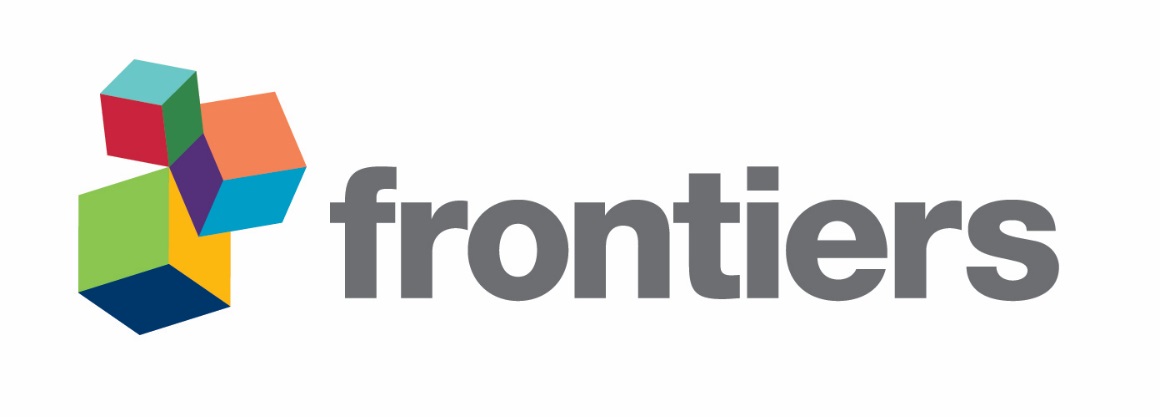
**
